# Reduced mitochondrial malate dehydrogenase activity has a strong effect on photorespiratory metabolism as revealed by ^13^C labelling

**DOI:** 10.1093/jxb/erw030

**Published:** 2016-02-17

**Authors:** Pernilla Lindén, Olivier Keech, Hans Stenlund, Per Gardeström, Thomas Moritz

**Affiliations:** ^1^Umeå Plant Science Centre, Department of Forest Genetics and Plant Physiology, Swedish University of Agricultural Sciences, SE-901 83 Umeå, Sweden; ^2^Umeå Plant Science Centre, Department of Plant Physiology, Umeå University, SE-901 87 Umeå, Sweden; ^3^Swedish Metabolomics Centre, Department of Molecular Biology, Umeå University, SE-901 87 Umeå, Sweden

**Keywords:** Heavy isotope labelling, mass spectrometry, mitochondrial malate dehydrogenase, photorespiration, primary carbon metabolism, redox balance.

## Abstract

Mitochondrial malate dehydrogenase (mMDH) catalyses the interconversion of malate and oxaloacetate (OAA) in the tricarboxylic acid (TCA) cycle. Its activity is important for redox control of the mitochondrial matrix, through which it may participate in regulation of TCA cycle turnover. In Arabidopsis, there are two isoforms of mMDH. Here, we investigated to which extent the lack of the major isoform, mMDH1 accounting for about 60% of the activity, affected leaf metabolism. In air, rosettes of *mmdh1* plants were only slightly smaller than wild type plants although the fresh weight was decreased by about 50%. In low CO_2_ the difference was much bigger, with mutant plants accumulating only 14% of fresh weight as compared to wild type. To investigate the metabolic background to the differences in growth, we developed a ^13^CO_2_ labelling method, using a custom-built chamber that enabled simultaneous treatment of sets of plants under controlled conditions. The metabolic profiles were analysed by gas- and liquid- chromatography coupled to mass spectrometry to investigate the metabolic adjustments between wild type and *mmdh1*. The genotypes responded similarly to high CO_2_ treatment both with respect to metabolite pools and ^13^C incorporation during a 2-h treatment. However, under low CO_2_ several metabolites differed between the two genotypes and, interestingly most of these were closely associated with photorespiration. We found that while the glycine/serine ratio increased, a concomitant altered glutamine/glutamate/α-ketoglutarate relation occurred. Taken together, our results indicate that adequate mMDH activity is essential to shuttle reductants out from the mitochondria to support the photorespiratory flux, and strengthen the idea that photorespiration is tightly intertwined with peripheral metabolic reactions.

## Highlight

Using a custom-built chamber for ^13^C labelling, we show that reduced mitochondrial malate dehydrogenase activity affects photorespiratory metabolism.

## Introduction

Mitochondrial malate dehydrogenase (mMDH) catalyses the interconversion of malate and oxaloacetate (OAA) in the tricarboxylic acid (TCA) cycle and its activity is important for redox control of the mitochondrial matrix, through which it may participate in regulation of the TCA cycle turnover (Nunes-Nesi *et al.*, 2005; Tomaz *et al.*, 2010). In addition, its activity is closely linked to malate/OAA exchange across the mitochondrial inner membrane. This process may be especially important for shuttling reductants between mitochondria and peroxisomes during photorespiration; in which equimolar amounts of NADH are produced during glycine oxidation in mitochondria and consumed in the reduction of hydroxypyruvate to glycerate in peroxisomes (Bauwe *et al.*, 2010). Also, by interplay with malate/OAA shuttling between chloroplasts and cytosol – the so-called ‘malate valve’– mMDH influences cellular redox balance (Scheibe, 2004). There are two isoforms of mMDH in *Arabidopsis thaliana*. Tomaz *et al.* (2010) found that the double mutant *mmdh1mmdh2* had a dramatically reduced growth rate, which could be, at least partly, related to disturbances in photorespiratory metabolism. Although the single mutants, *mmdh1* and *mmdh2*, both had a reduced mMDH activity (~60% and ~40% reductions, respectively), neither displayed an apparent growth phenotype when grown under a controlled environment in air. Nonetheless, a slight perturbation in the CO_2_ response curve was recorded in *mmdh1*, suggesting that photorespiration might also be affected in these plants (Tomaz *et al.*, 2010).

Metabolite analysis can provide good indications of an organism’s physiological status at a given time under given conditions. However, such snapshots of metabolites’ pool sizes can be complemented by metabolic fluxes and dynamics analyses in order to provide additional information and better decipher an organism’s metabolic responses to environmental changes (Wiechert *et al.*, 2007; Sweetlove *et al.*, 2014). A well-established method for flux analysis in plants is feeding a ^13^C-labelled substrate to a cell suspension culture until a steady state is reached (Wiechert, 2001; Wittmann and Heinzle, 2002; Sauer *et al.*, 2004; Matsuda *et al.*, 2007; Williams *et al.*, 2008). ^13^C is a favourable tracer as it is safe to handle, clean, stable, and compatible with mass spectrometry (MS) and nuclear magnetic resonance spectroscopy (NMR) techniques (Fernie *et al.*, 2005). However, for flux analysis in non-steady state systems, such as photosynthetic leaf tissue, the steady-state ^13^C analysis provides little information and requires additional strategies (Sweetlove *et al.*, 2014). Determining kinetics of labelled metabolites in non-steady state systems remains challenging, and several ^13^CO_2_-based approaches have been proposed to address metabolic fluxes *in planta* (Huege *et al.*, 2007; Arrivault *et al.*, 2009; Szecowka *et al.*, 2013; Heise *et al.*, 2014; Ma *et al.*, 2014). Another hurdle for ^13^C analyses in plants lies in the lack of appropriate labelling chambers that allows control of the ^13^CO_2_ concentration and growth conditions, while facilitating rapid sampling and freezing of exposed leaves, without disturbing their environment. These features are essential for accurate determinations of pool sizes, their dynamics and robust conclusions regarding metabolic regulation. Furthermore, metabolite analysis is challenging, regardless of labelling, due to the sheer number of metabolites, the diversity of their chemical properties and the huge concentration differences (Fiehn, 2002; Weckwerth, 2011). Nonetheless, coverage can be improved by using multiple analytical techniques, for example, liquid- or gas chromatography coupled with MS (LC- and GC-MS) and NMR. Such combination analysis can substantially increase metabolite detection, thereby improving the depth of biological understanding (t’Kindt *et al.*, 2009; Hiller *et al.*, 2011; Kueger *et al.*, 2012; Szecowka *et al.*, 2013; Heise *et al.*, 2014).

Here we report experiments using a specially constructed chamber for exposure of plants to ^13^CO_2_ under controlled conditions. A set of plants, consisting of both wild type and *mmdh1*, were subject to high and low CO_2_ treatments, and sampled before and after 30, 60 and 120min of ^13^C labelling. Forty metabolites were detected with LC and GC-MS techniques to compare metabolic adjustments between wild type and *mmdh1* plants under reduced or high photorespiratory conditions.

## Materials and methods

### Plant material

Wild type *Arabidopsis thaliana* (ecotype Columbia-0) and the T-DNA insertion line *mmdh1* (Tomaz *et al.*, 2010) seeds were sown on 1:4 perlite:soil (Hasselfors Garden, P-jord; NPK 14:7:18, pH 6, magnesium 250g m^−3^). After stratification (+4°C, 48h), seeds were transferred to a growth chamber, under short-day conditions: 8h light (22°C)/16h dark (17°C), and 75% relative humidity and with a light intensity of 180 µmol m^−2^ s^−1^ photosynthetically active radiation (PAR). Once a week the pots were randomized between trays to avoid systematic bias in growth arising from variations in microclimate within the chamber.

Growth phenotype was assessed from 7-week-old plants, which were placed in either ambient CO_2_ (i.e. 380 ppm) or in low CO_2_ (i.e. 150 ppm) conditions for 6 weeks.

### Chemicals

^13^CO_2_ (25 l bottle; isotopic purity 90 atom % ^13^C, <1.5 atom % ^18^O) purchased from Spectra products (Littleport, Cambridgeshire, UK) and ^12^CO_2_ (20 l bottle) from AGA (Sweden). Atmospheric air was tapped from an internal supply. All metabolite standards and other chemicals were purchased from Sigma-Aldrich (Minneapolis, MN, USA), except sedoheptulose-7-phosphate, which was bought from Carbosynth Ltd (Berkshire, UK). All standards were purchased at the highest available purity.

### Chamber construction

The chamber was built in a glove-box style (Supplementary Fig. S1, available at *JXB* online). The enclosure housing the plants was custom-built by Rexonic AB (Piteå, Sweden) from 8mm thick Plexiglas (volume 0.12 m^3^). It was mounted on a metal scaffold, above a table-top surface, leaving space for a freezing container and providing an ergonomically comfortable working height. The scaffold also supported the light source; a high-pressure metal-halide lamp providing a light intensity of 180 μE m^−2^ s^−1^ at plant level. A pair of integrated rubber gloves enabled sampling during treatment. An extraction port with a membrane in the chamber floor allowed fast transfer of samples into a container with liquid nitrogen. The extraction port was strategically placed to minimize risks of shading the plants when working in the chamber. An expansion vessel compensated for the reduction in air volume resulting from hands working in the chamber. A one-way restrictor valve allowed the release of internal pressure during treatment. The gas system included three lines (coupled to the chamber via a single merged inlet port). One line was for nitrogen (humidified before entering the chamber) to flush CO_2_ from the chamber prior to treatments. One supplied ^13^CO_2_ via a regulator fitted with a magnetic valve, controlled by a custom-made computer interface, after mixing with CO_2_-free air (both ^12^CO_2_ and ^13^CO_2_ gas could be used in ranges from 100 to 10 000 µl l^−1^). The other was the air supply, tapped from the laboratory’s gas lines and controlled by a high-pressure regulator and a rotameter (Platon NG, Type FNGVB211A, Roxspur Measurement & Control Ltd, Sheffield, UK). CO_2_ was removed from the air by two aqueous CO_2_ scrubbers in series followed by a limestone cartridge.

The cooling system consisted of a heat exchange package through which water was pumped continuously by a F12-MA thermoregulated water bath (Julabo GmbH, Seelbach, Germany). Two fans, controlled by the computer interface, were placed behind the heat exchanger. The fans were switched on if the temperature exceeded the set target value. An additional fan for air circulation was kept running when the chamber was in use.

An Engine K30 FR CO_2_ sensor (SenseAir, Delsbo, Sweden) was used to monitor CO_2_ levels and a Sensirion SHT75 dew point sensor (Sensirion, Staefa ZH, Switzerland) was used to monitor temperature and relative humidity. Both sensors were connected to an Arduino UNO microcontroller (http://arduino.cc/), to enable communication with the computer interface. The computer interface was programmed using MATLAB ver. 8.1 (MathWorks, Natick, MA, USA). The data collected by the sensors were displayed by the interface in real-time plots (^12^CO_2_, ^13^CO2, temperature and RH) and data were automatically recorded as *.txt files.

### Plant treatments

The temperature was kept at 22 °C in the labelling chamber, and the relative humidity at 80–85%. The ^13^CO_2_ gas pressure was 1 bar and the air flow rate to the CO_2_ scrubber was 2 l min^−1^ for the high CO_2_ treatment and 5 l min^−1^ for the low CO_2_ treatment. Target values for the high and low CO_2_ treatments were 1 000 and 150 µl l^−1^, respectively. All experiments were initiated in the middle of the photoperiod.

### Sampling

Fully expanded leaves were cut from 6-week-old Arabidopsis plants in the labelling chamber, transferred to a 20ml scintillator tube that was loosely capped (making it possible for the liquid nitrogen to come in contact with the sampled leaf) and dropped into liquid nitrogen through the extraction port (the whole sampling procedure took under 10s). Four biological replicates (i.e. independent plants) per genotype were sampled at four time points: just before treatment and after 30, 60 and 120min treatment.

### Metabolite extraction and derivatization

Samples (19–21mg frozen and ground leaf material) were extracted according to Gullberg *et al.* (2004). In brief, stable isotope reference compounds (7ng μl^−1^ [^13^C_3_]-myristic acid, [^13^C_4_]-hexadecanoic acid, [^2^H_7_]-cholesterol, [^13^C_5_]-proline, [^2^H_4_]-putrescine and [^2^H_6_]-salicylic acid) were added to a chloroform:methanol:water (20:60:20, v/v/v) extraction mixture. 1ml of the spiked mixture was added to each sample in 1.5ml tubes (Sarstedt, ref: 72.690.007) on ice. After adding a 3mm tungsten carbide bead (Retsch GmbH & Co. KG, Haan, Germany) to each tube they were shaken at 30 Hz for 3min in a MM 301 Vibration Mill (Retsch GmbH & Co. KG, Haan, Germany). The beads were removed before centrifugation for 10min at 14 000rpm in a Mikro 220R instrument (Hettich, Zentrifugen). The supernatant from each tube (200 μl) was transferred to a 250 μl micro vial (Chromatol Ltd) and evaporated to dryness in a miVac quattro concentrator (Barnstead genevac). Samples were derivatized by adding 30 μl methoxyamine hydrochloride (15mg ml^−1^) in pyridine and shaking for 10min at 5°C in a VX-2500 Multi-tube Vortexer (VWR Scientific), followed by 16h incubation at room temperature, then adding 30 µl MSTFA in 1% TMCS for silylation, vortex-mixing, then 1h incubation at room temperature. Heptane (30 μl, including 15ng μl^−1^methyl stearate) was added and the samples were ready for MS analysis after vortex-mixing.

### LC-MS analysis

Analysis was done by combined ultra-high-performance liquid chromatography- electrospray ionization-triple quadrupole-tandem mass spectrometry (UHPLC-ESI-QqQ-MS/MS) in multiple-reaction-monitoring (MRM) mode. An Agilent 6490 UHPLC chromatograph equipped with a Waters Acquity UPLC BEH Amide1.7 µm, 2.1×50mm column (Waters Corporation, Milford, USA) coupled to a QqQ-MS/MS (Agilent Technologies, Atlanta, GA, USA) was used. The washing solution, for the auto sampler syringe and injection needle, was isopropanol:water (1:1, v/v). The mobile phase consisted of 85% B (acetonitrile:10mM aqueous ammonium formate, v/v) for 0.5min followed by linear gradients from 85 to 70% from 0.5 to 5.5min then 70 to 10% B from 5.5 to 8min, followed by 85% B for equilibration from 8 to 15min. The balance (mobile phase A) consisted solely of 10mM aqueous ammonium formate. The flow rate was 1.6 l min^−1^ during equilibration and 0.250 l min^−1^ during the chromatographic runs. The column was heated to 60°C, and injection volumes were 2 μl. The mass spectrometer was operated in negative ESI mode with gas temperature 210°C; gas flow 11 l min^−1^; nebulizer pressure 60 psi; sheath gas temperature 200°C; sheath gas flow 8 l min^−1^; capillary voltage 3 000V (neg.); nozzle voltage 0V; iFunnel high pressure RF 90V; iFunnel low pressure RF 60V. All MRM transitions were run in negative mode: dwell time 50s; fragmentor voltage 380V; cell acceleration voltage 5V. Every sample was injected twice to reduce the number of MRM transitions per analysis. For a list of MRM transitions see Supplementary Table S1. Data were normalized with respect to sample fresh weights and processed using MassHunter Qualitative Analysis and Quantitative Analysis (QqQ; Agilent Technologies, Atlanta, GA, USA) and Excel (Microsoft, Redmond, Washington, USA) software.

### GC-MS analysis

The GC-MS analysis followed the GC-TOF-MS procedure published by Gullberg *et al.*, (2004). Electron inpact (EI) was used for ionization. Quality control samples and a n-alkane series (C_8_–C_40_) were included in each analysis ((Schauer *et al.*, 2005). The derivatized samples (1 μl) were injected into a split/splitless injector in splitless mode, by an CTC PAL systems auto sampler (with a 10 μl syringe), into an Agilent technologies 7890A GC system (Agilent Technologies, Atlanta, GA, USA) equipped with a 30 m×0.250mm diameter fused silica capillary column with a bonded 0.25 μm Durabond DB-5MSUI stationary phase (part no. 122-5222UI, Agilent J&W GC columns). The settings were: injector temperature, 260°C; front inlet septum purge flow rate, 3ml min^−1^; gas flow rate, 1ml min^−1^; column temp 70°C for 2min, then increased by 20°C min^−1^ to 320°C (held for 8min). The column effluent was introduced into the ion source of a Pegasus HT GC, high-throughput TOF-MS (LECO Corp., St Joseph, MI, USA), with: transfer line temperature, 270°C; ion source temperature, 200°C; detector voltage, 1520V; electron impact electron beam, −70V; ionization current, 2.0 mA. 20 spectra s^−1^ were recorded with a 50–800 m/z mass range, and 290s solvent delay.

The raw data were converted from SMP-format to NetCDF-format using ChromaTOF software. Peak detection and peak area calculations of both labelled and unlabelled fragments (selected fragments listed in Supplementary Table S2) were performed using Frag_calc, in-house software programmed in MATLAB ver. 8.1 (MathWorks, Natick, MA, USA). Frag_calc required a text file as input, containing unique names, ion channels and retention time windows of the metabolites to be analysed. Data were normalized with respect to internal standards according to (Redestig *et al.*, 2009). Unlabelled metabolites were identified by comparing their retention indices and mass spectra with entries in commercial and in-house mass spectra libraries using NIST MS Search 2.0 (National Institute of Standards and Technology, 2001). In-house software, 13C_est, was used to correct for natural abundance of ^13^C and isotope contributions from TMS-groups, and to calculate percentages of ^13^C incorporation for each identified metabolite.

### Statistical analysis

Multivariate analysis was performed using SIMCA 13.0 software (Umetrics, Umeå, Sweden). All variables were log_10_-transformed, mean-centred, and scaled to unit variance before further analysis. Principal component analysis (PCA) was used to overview the data, e.g. observe trends/clusters and detect outliers. Orthogonal projection to latent structures (OPLS) analysis, a supervised technique, was used to connect information regarding two-block variables (X and Y) (Trygg and Wold, 2002) and OPLS-Discriminant (OPLS-DA) analysis was used for modelling maximum class separation (Trygg and Wold, 2002; Bylesjö *et al.*, 2006; Trygg *et al.*, 2006). For all models R2X(cum) is the cumulative modelled variation in X, while R2Y(cum) is the cumulative modelled variation in Y. The range of these parameters is 0–1, where 1 indicates a perfect fit. Q2 is the estimated predictive ability of the model (−1 to 1).

## Results

**Fig. 1.**
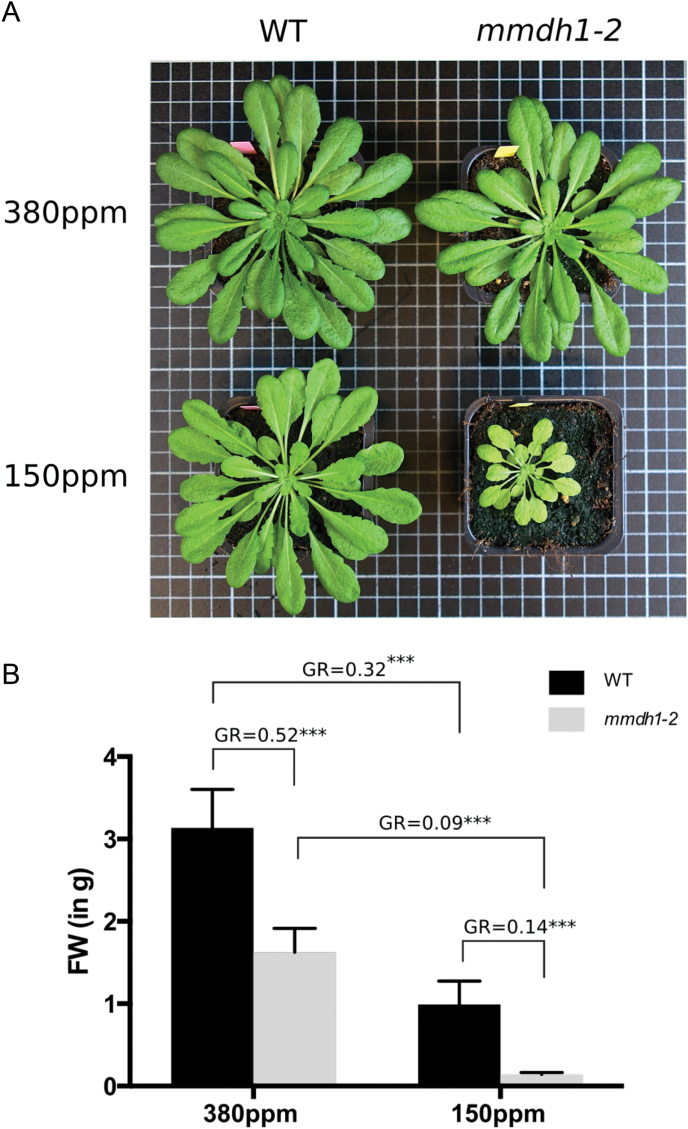
(A) Phenotypic differences between 7-week-old wild-type and *mmdh1* mutant plants grown under either ambient air (380 ppm) or low CO_2_ (150 ppm) conditions. (B) Shoot biomass quantification (FW, fresh weight) for plants described in panel A with *n*=7; GR, growth ratio. Significance *** refers to a *P*<0.001 with a Sidak’s multi-comparison test in a two-way ANOVA.

It has been reported that when grown under ambient air conditions, the two single mMDH mutant plants, i.e. *mmdh1* and *mmdh2*, did not exhibit apparent growth phenotype (Tomaz *et al.*, 2010). Still, a slight perturbation in the CO_2_ response curve was observed, suggesting that photorespiration might be affected. Here, wild-type and *mmdh1* plants were grown under short day conditions and in CO_2_ controlled environments for 6 weeks. Under 380 ppm CO_2_, the rosettes of *mmdh1* were only slightly smaller than the ones from wild-type plants while a drastic growth difference was noticeable when plants were grown under 150 ppm CO_2_ (Fig. 1A). In addition, the respective shoot biomass fresh weight was quantified. The growth ratio (GR) between wild type and *mmdh1* was estimated at nearly 50% under 380 ppm CO_2_ whereas *mmdh1* produced only 14% of the wild-type shoot biomass under low CO_2_ conditions (Fig. 1B). Interestingly, the difference in shoot biomass production between ambient air and low CO_2_ conditions was 68% for wild-type plants whereas it reached more than 90% in the *mmdh1* mutant plants. Together, this phenotypical quantification clearly showed that a reduced amount of mMDH affects plant growth, particularly under high photorespiratory conditions. This therefore prompted us to investigate in more detail the metabolic adjustments in *mmdh1*, particularly under high and low photorespiratory conditions.

### Validation of the labelling chamber

**Fig. 2.**
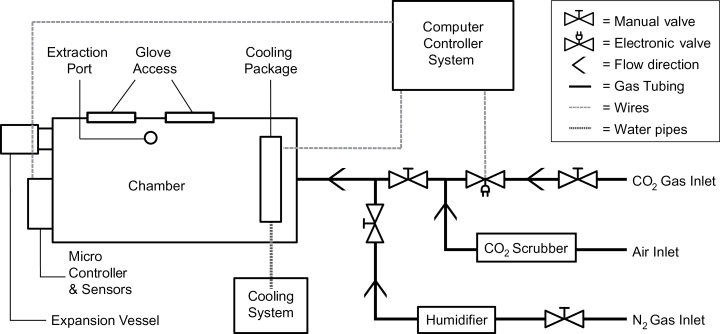
Schematic diagram of the labelling chamber and peripheral equipment, including the gas, cooling and control systems.

A labelling chamber, designed as a glove box, was constructed with the capacity to house eight fully-grown Arabidopsis plants. The chamber enabled simultaneous treatment of four biological replicates of two genotypes, but also handling and sampling plants during treatment (Fig. 2). A connected gas system provided either ^12^CO_2_ or ^13^CO_2_ at controlled concentrations. Samples were collected within the chamber, and directly snap frozen by pushing the sample tubes through an extraction port in the chamber floor into a liquid nitrogen bath. Sampling did not disturb the experimental environment.

**Fig. 3.**
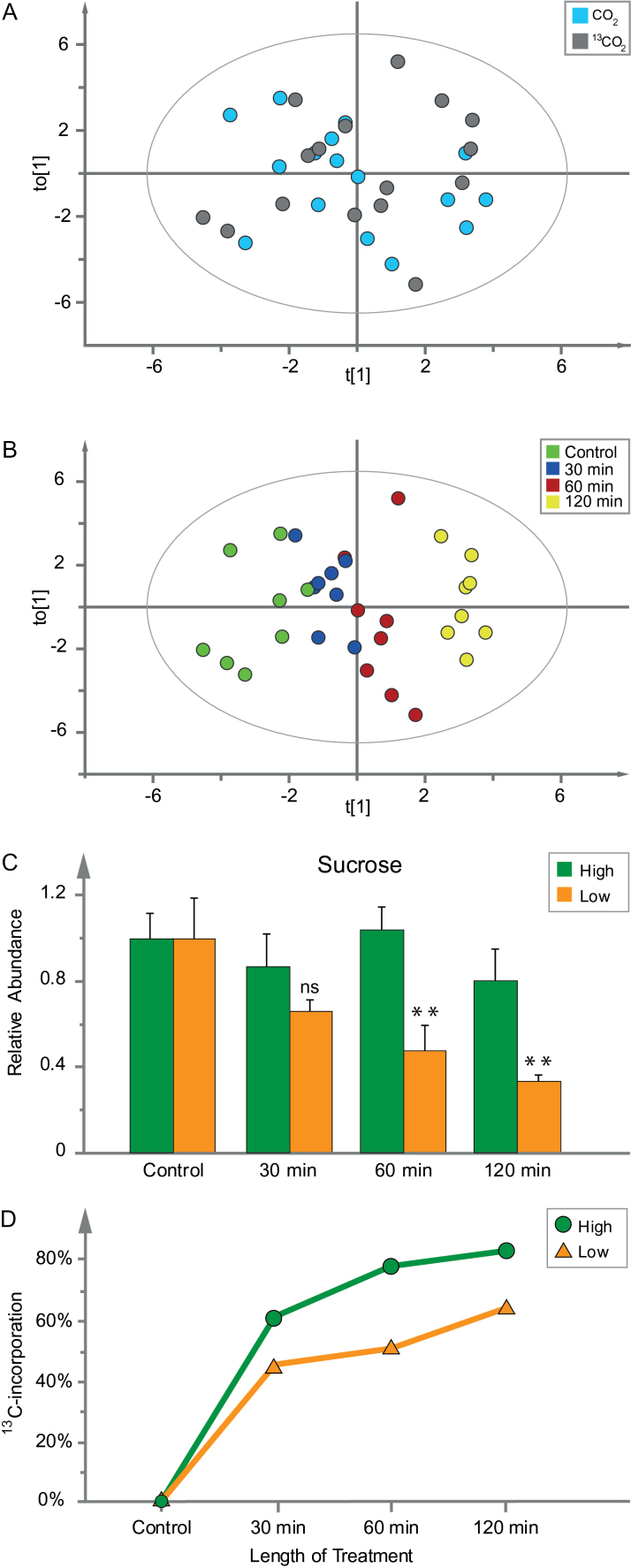
Chamber validation by two experiments with wild-type plants in low CO_2_ (one unlabelled and the other with ^13^CO_2_). (A) OPLS score plot, to[1]/t[1], confirming there was no clear separation of results between treatment with CO_2_ (blue) and ^13^CO_2_ (grey). (B) The same score plot as in panel A, but coloured by time points: control (green), 30min (blue), 60min (red), 120min (yellow) (OPLS model: components=1+1, Y-variable=time, X-variables=37, *n*=32, R2X(cum)=0.317, R2Y(cum)=0.911, Q2=0.791). (C) Validation using data for relative abundance of sucrose (scaled to control). Green bars represent an average of four samples treated in high CO_2_ conditions and orange bars represents an average of four samples treated in low CO_2_ conditions. Relative abundance of sucrose under high and low CO_2_ conditions was compared by a Student’s *t*-test and significance was marked as ** for a *P*<0.01. (D) ^13^C incorporation in sucrose, in leaves of wild-type Arabidopsis plants exposed to high (green circles) and low (orange triangles) CO_2_ treatment over 2h. Data were normalized to fresh weight (mg) and technical variation (t-score of internal standards).

The chamber’s ability to provide reproducible conditions and uniformly exposed plants to the ^13^C tracer was tested in four preliminary experiments by four sets of wild-type Arabidopsis plants. The CO_2_ concentration was kept at 1 000 µl l^−1^ in two of these experiments and at 150 µl l^−1^ in the other two (hereafter referred to as ‘high’ and ‘low’ CO_2_, respectively), in both cases once with ^12^CO_2_ and once with ^13^CO_2_. Fully grown leaves were sampled before treatment and after 30, 60 and 120min of each treatment, then analysed by GC-MS. Data acquired from the experiments were compared by OPLS (Trygg *et al.*, 2006), using length of treatment as the Y-variable and metabolite abundances as the X-variables. A t_o_[1]/ t[1] score plot including all four experiments showed the samples based on the variation in X depending on Y, the orthogonal variation was the CO_2_ concentration (Supplementary Fig. S2). The results showed that the plants responded to the treatments and validated the chamber’s suitability for exposing plants to varied CO_2_ concentrations. The reproducibility between experiments was also evaluated by OPLS, by comparing two experiments with the same CO_2_ concentration. The score plot from the model of the low CO_2_ treatment showed no separation between experiments (Fig. 3A), but consistent separation of time points (Fig. 3B). As a final validation of the robustness of the chamber an OPLS model of data from each low CO_2_ experiment was used to predict the metabolic profile of the samples from the other low CO_2_ experiment. The responses proved to be highly linear, with R2-values of 0.95 and 0.61, showing that the chamber provided highly reproducible conditions (Supplementary Fig. S3A, B).

To scrutinize the ^13^C incorporation we measured the abundance and ^13^C labelling of sucrose under high and low CO_2_ conditions. Sucrose was chosen since it is a stable product from carbon fixation. Sucrose production was expected to be reduced in low CO_2_ treatment compared to high CO_2_ treatment. Indeed, the relative abundance of sucrose decreased during the 2-h low CO_2_ treatment, but remained stable in the high CO_2_ treatment (Fig. 3C). Furthermore, sucrose was more rapidly labelled in high CO_2_ treatment compared to low (Fig. 3D). Hence, we concluded that the ^13^CO_2_ labelling of plants in the chamber was robust under both high and low CO_2_ treatments.

### Metabolite detection and calculation of ^13^C incorporation

To detect ^13^C incorporation in metabolites early in carbon fixation, such as hexose phosphates (fructose-6-phosphate, glucose-6-phosphate and glucose-1-phosphate) and UDP-glucose, the LC-MS system was operated in negative MRM mode. The pseudomolecular ion of each compound, [M-H^−1^], was set as the precursor ion and the phosphate group as the product ion ([PO_3_]^−^ m/z 79 or [PO_4_]^−^ m/z 96). For each labelled metabolite there are (*n*+1) possible isotopomers, were *n* is the number of carbons in the metabolite (conceptual illustration Supplementary Fig. S4A). Hence, (*n*+1) precursor ions were detected for every labelled metabolite. In unlabelled plant material 12 additional sugar phosphates were detected, but their abundance was too low for estimation of ^13^C incorporation.

The majority of the metabolites were identified by GC-TOF-MS after methoxyamine and trimethylsilyl (TMS) derivatization. To calculate the ^13^C incorporation correctly the contribution from the derivatization and the number of carbons in the fragment must be known. For example, the most abundant fragment for the two carbon metabolite glycine is m/z 276. This fragment contains both carbons and three TMS-groups where one of the TMS-groups has lost a methyl group. Once this relation has been established the (*n+*1) formula can be used to decide the number of isotopomers to be detected. Thus, for ^13^C-labelled glycine m/z 276 to 278 must be monitored to be able to calculate the ^13^C incoporation correctly (conceptual illustration Supplementary Fig. S4B). Hence, the number of isotopomers required to cover the ^13^C incorporation increases with the number of carbons of the metabolite and the total number of isotopomers of the analysis increases with the number of metabolites analysed.

**Fig. 4.**
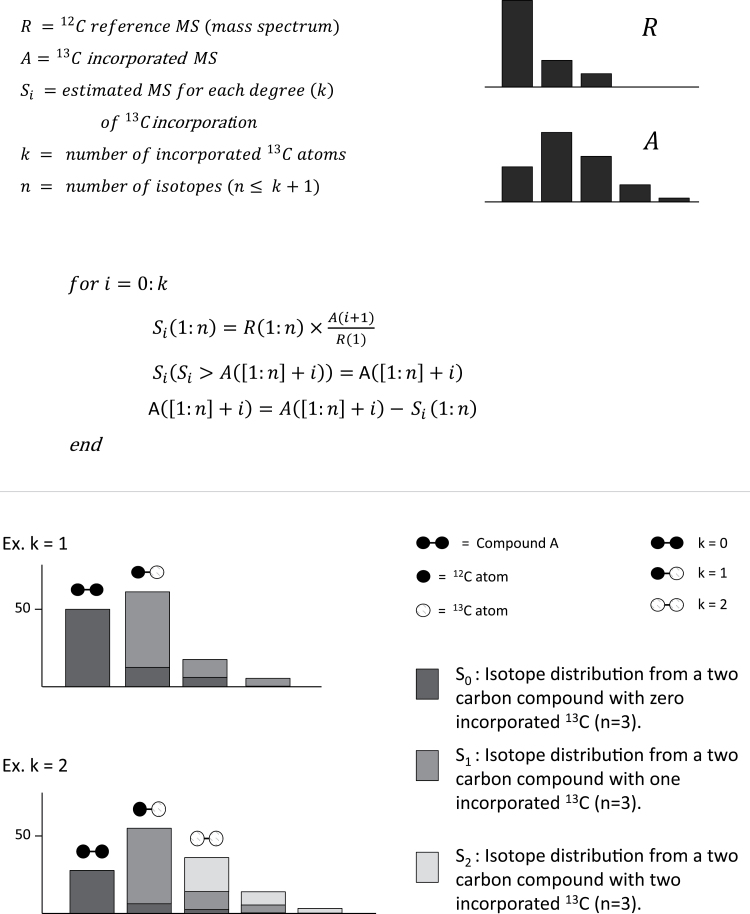
Principle of ^13^C estimations. The ^13^C incorporation was calculated by sequential isotope compensation from a reference spectra (unlabelled).

Metabolite abundance and ^13^C incorporation were calculated by a targeted approach, were a list of all metabolite fragments were processed by in-house scripts. In the same process the natural occurrence of ^13^C, ~1.1% of all C carbons (Smith, 1972), and the contribution from the TMS-groups (for the GC-MS data), was subtracted. This was done by sequential isotope compensation from an unlabelled reference spectrum (Fig. 4). A visualization plot was generated for every metabolite, showing the calculated percentage incorporation for every isotope, the relative standard deviation (RSD) of the unlabelled control, and the contributions from the natural abundance of ^13^C and the TMS-groups (Supplementary Fig. S5). The visualization plots were an important part of the quality evaluation of the data.

### Comparison of metabolic phenotypes of wild type and mmdh1

**Fig. 5.**
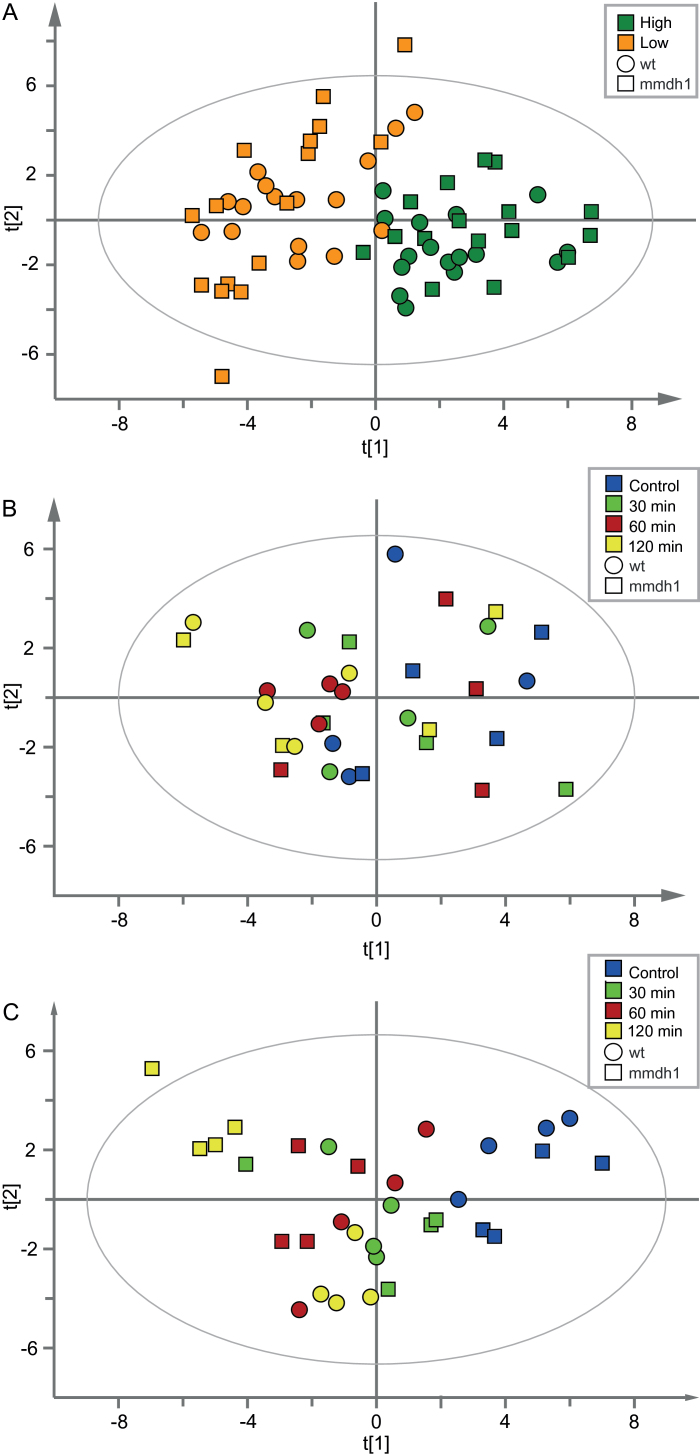
PCA score plots for *mmdh1* and wild type leaves, under: (A) high (dark green) and low (orange) CO_2_ treatment (PCA model: components=5, *n*=64, X-variables=37, R2X(cum)=0.731, Q2=0.455); (B) high CO_2_ treatment (PCA model: components=4, *n*=32, X-variables=37, R2X=0.623, Q2=0.187); (C) low CO_2_ treatment (PCA model: components=4, *n*=32, X-variables=37, R2X=0.701, Q2=0.402). The colour coding indicates sampling times: control (blue), and after 30min (green), 60min (red) and 120min (yellow) treatment. Data were normalized to fresh weight (mg) and technical variation (t-score of internal standards).

Metabolic profiles of wild type and *mmdh1* under different CO_2_ concentrations, using ^13^CO_2_, were acquired by GC- and LC-MS analyses and first explored by PCA. Score plots for samples of both genotypes exposed to high and low CO_2_ showed a clear separation between treatments, but no evident differences between genotypes (Fig. 5A). A second PCA model based solely on data from samples (wild type and *mmdh1*) exposed to high CO_2_ showed no clear differences between genotypes, or changes over time (Fig. 5B). However, a PCA model based on metabolic profiles of the two genotypes at low CO_2_ showed a progressive separation of the genotypes during the time course (Fig. 5C). This was further confirmed by OPLS-discriminant analysis (OPLS-DA) (Trygg *et al.*, 2006), which showed significant correlations between shifts in abundance of some metabolites and one or the other of the genotypes. Under low CO_2_, the mutant displayed a time course separation in the first orthogonal component (vertical axis of the OPLS-DA score plot; Supplementary Fig. S6C). Collectively, these results clearly indicated that metabolic differences between the *mmdh1* mutant and wild type were most pronounced under low CO_2_ concentrations, while the two genotypes responded very similarly under high CO_2_.

**Fig. 6.**
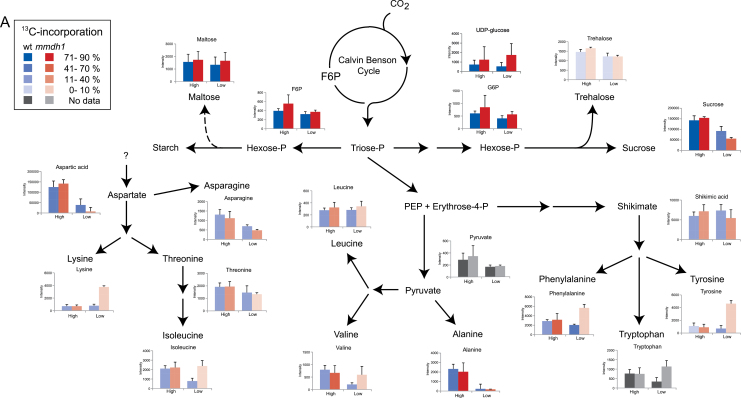

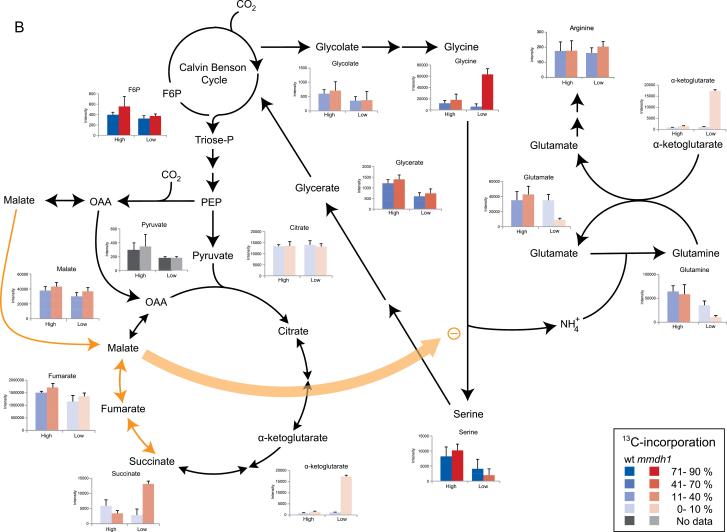
(A) Summary of metabolite abundance and ^13^C incorporation from the Calvin-Benson cycle to amino acids and sugars. Bar graphs show metabolite abundance in wild-type (blue) and *mmdh1* (red) samples after 2h treatment under high or low CO_2_ conditions. The opacity (darkness of shading) indicates the degree of ^13^C incorporation. (B) Summary of metabolite abundance and ^13^C incorporation from the Calvin-Benson cycle to the TCA cycle and photorespiratory pathway. Bar graphs show metabolite abundance in wild-type (blue) and *mmdh1* (red) samples after 2h treatment under high or low CO_2_ conditions. The opacity (darkness of shading) indicates the degree of ^13^C incorporation. Orange arrows indicate the proposed imbalance of reducing equivalents caused by mMDH1 deletion.

More specifically, after 2h treatment in low CO_2_ treatment the levels of sucrose, two organic acids (pyruvate and glycerate) and several amino acids (asparagine, aspartate, alanine and serine) had decreased in both genotypes (Table 1, Fig. 6, Supplementary Tables S3–6). Interestingly, levels of isoleucine, valine, tryptophan, phenylalanine and tyrosine decreased in wild-type plants, while levels of glutamate and glutamine decreased in the *mmdh1* mutant. In addition, in *mmdh1* plants, pools of several metabolites (succinate, α-ketoglutarate, lysine, glycine, and the three aromatic amino acids: tyrosine, tryptophan and phenylalanine) were more abundant under low CO_2_ than under high CO_2_, a relation that was not found in the wild type counterparts (Fig. 6). Thus, due to the increase in glycine and decrease in serine content in low CO_2_, the glycine/serine ratio was much higher in *mmdh1* than in wild-type plants (Supplementary Fig. S7). In high CO_2_ the glycine/serine ratio was low in both mutant and wild type whereas the ratio in ambient CO_2_ was intermediate between high and low CO_2_ in the mutant. In wild type under limiting CO_2_ the glycine/serine ratio initially increased during the first hour but then returned to a low ration after 2h, suggesting an adaptive mechanism.

**Table 1.** ^13^C incorporation (%) in detected metabolites in wild-type (W) and *mmdh1* (M) samples under high (>1 000 ppm) and low (200 ppm) CO_2_. T1, 30min treatment; T2, 60min treatment; T3, 120min treatment. Values should be considered as indicators rather than exact quantitative values. *, missing value.

	High CO_2_^13^C (%)						Low CO_2_^13^C (%)					
	T1		T2		T3		T1		T2		T3	
	W	M	W	M	W	M	W	M	W	M	W	M
a-ketoglutaric acid	4	1	9	-	10	6	7	-	1	-	1	-
Alanine	52	55	63	62	67	73	41	51	31	59	55	50
Arginine	14	23	18	25	36	40	20	20	18	26	32	28
Ascorbic acid	-	-	3	1	10	6	3	2	4	1	1	1
Asparagine	2	5	9	18	23	33	8	10	12	22	28	33
Aspartic acid	41	40	48	50	60	65	32	31	34	26	47	37
β-alanine	-	3	1	-	-	-	6	-	4	-	-	-
Citric acid	-	1	1	1	1	2	1	-	-	-	-	1
Fructose	20	10	26	24	36	42	8	9	10	16	23	20
Fumaric acid	3	3	5	7	12	14	4	4	5	5	8	10
GABA	9	5	25	9	16	19	29	-	13	6	8	18
Glucose	8	5	11	11	14	17	4	5	5	5	6	8
Glutamic acid	4	6	8	10	13	15	1	1	3	2	3	3
Glutamine	3	5	6	9	12	15	2	2	2	1	3	4
Glyceric acid	45	46	54	56	64	64	39	45	48	41	53	50
Glycerol	1	-	-	1	1	2	-	2	-	1	1	1
Glycine	27	25	50	56	49	65	41	71	57	72	57	72
Glycolic acid	22	16	29	22	31	30	19	25	28	24	19	24
Isoleucine	23	24	25	32	32	40	9	11	12	9	30	-
Leucine	33	30	20	35	26	32	-	-	5	-	37	-
Lysine	25	28	19	31	23	29	9	6	14	6	23	-
Malic acid	8	7	9	11	17	18	9	9	11	11	19	15
Maltose	82	78	83	80	87	90	77	75	74	73	81	74
Phenylalanine	26	23	26	34	35	43	18	23	20	26	41	8
Serine	76	74	81	82	88	90	67	66	70	66	82	67
Shikimic acid	12	13	19	21	28	32	10	12	15	13	18	12
Spermidine	-	-	-	1	4	-	4	-	3	-	6	1
Succinic acid	1	7	1	12	2	17	-	17	-	15	1	14
Sucrose	60	53	76	74	81	84	42	44	47	56	59	57
Threonine	2	5	5	8	16	24	5	7	5	11	16	10
Trehalose	8	6	6	5	4	7	6	3	10	4	9	9
Tyrosine	17	13	NA*	25	8	25	-	-	2	4	22	-
Valine	23	25	25	33	39	43	11	10	9	20	37	NA*

Moreover, the ^13^C incorporation estimations showed that the metabolites directly produced from and closely associated with the Calvin-Benson cycle, were rapidly labelled. This was exemplified by sucrose and maltose, as well as metabolites associated with photorespiration, such as glycine, serine and glyceric acid (Table 1, Fig. 6). Accordingly, hexose-phosphates were rapidly and massively labelled (more than 70% incorporation after 30min; Supplementary Fig. 8). Glycolate was also labelled albeit more weakly than the other analysed photorespiratory intermediates (in both genotypes at every time point and under both CO_2_ treatments). Generally, ^13^C-incorporation patterns were similar in mutant and wild-type samples under both high and low CO_2_ conditions (Table 1). Apart from glycine, most of the amino acids were more strongly labelled under high than low CO_2_ conditions. Several amino acids were rapidly labelled, including: aspartate and its biosynthetic derivatives isoleucine and lysine; the aromatic amino acid phenylalanine, for which the biosynthetic intermediate shikimate also showed substantial labelling; and both alanine and valine, which are linked to pyruvate metabolism. Unfortunately, the abundance of pyruvate was too low for a reliable detection of ^13^C incorporation. At most time points glycine was more strongly labelled in *mmdh1* than in wild-type plants, particularly under low CO_2_. Intriguingly, glutamate and glutamine were slightly labelled under high CO_2_, but hardly at all under low CO_2_ (Table 1). This is interesting as they are key intermediates in refixation of the NH_4_^+^ released in mitochondria during photorespiratory conversion of glycine to serine. Several organic acids (e.g. fumarate and malate) showed an intermediate labelling rate. Other metabolites, including citrate and α-ketoglutarate, showed a very low labelling rate. Finally, uniquely among the monitored metabolites, substantial incorporation of ^13^C was detected for succinate in *mmdh1*, but not in wild-type plants.

## Discussion

### ^13^C labelling and detection techniques for metabolite analysis

The labelling chamber, developed and applied in this study, offers unique possibilities to expose plants to ^13^CO_2_, with satisfactory numbers of biological replicates. The chamber was easy to handle as a set of plants could be exposed to ^13^CO_2_ at a desired concentration, ranging from 100 to 10 000 µl l^−1^, under carefully controlled and recorded conditions. Nonetheless, the chamber had one main limitation: its large volume would prevent rapid atmospheric changes, and hence high-resolution kinetic analysis of metabolic responses. It is however unlikely that this would strongly affect metabolites other than those with a fast turnover.

The aim during development of the analytical methodology was to combine a fast sample preparation with quick LC- and GC-MS methods enabling high-throughput analysis and providing good coverage of plant primary carbon metabolism. Thus, the same extraction protocol was used for both MS analyses (apart from the derivatization step for the GC analysis), which inevitably prevented detection of some metabolites that would have required a more elaborate extraction. This resulted in a relatively low coverage of metabolites involved in early stages of carbon assimilation, but not for metabolites acting further downstream in the plants’ primary metabolism. For the GC-MS analysis the major challenge in label detection was to find the most representable fragment for each metabolite, e.g. containing the highest possible number of carbons without interference from other compounds, preferably with a high signal-to-noise ratio. The use of customized in-house scripts dramatically reduced the time required for data processing and aided both interpretation of the results and quality control.

### mmdh1 has photorespiratory perturbations resulting from a redox imbalance

The responses to high CO_2_ treatment were very similar between wild-type and *mmdh1* plants, both with respect to metabolite pools and incorporation of ^13^C (Fig. 6). Also, when high and low CO_2_ were compared, the differences observed were in most cases similar between wild type and mutant including decreased pools of hexoses and sucrose, and some amino acids (aspartate, asparagine and alanine). However, under low CO_2_ treatment both abundance and labelling of some metabolites significantly differed between the two genotypes. Interestingly, most of the observed differences were closely associated with photorespiration. The glycine/serine ratio increased in mutant as compared to wild-type plants and the effect was more pronounced at low CO_2_ as compared to ambient air and high CO_2_ (Supplementary Fig. S7). The increase in glycine/serine ratio could indicate that in *mmdh1* the limitation in OAA to malate conversion directly influences the glycine to serine conversion although the increased ratio could also reflect an adjustment to maintain the flux through the GDC. However, the significant reduced growth of mutant plants in strong photorespiratory conditions and the effects on glutamate/glutamine/α-ketoglutarate (see below) support a direct limitation in the reaction. A reduced capacity to shuttle NADH produced in glycine decarboxylation from the mitochondria out to the peroxisomes is likely to result in an increased NADH/NAD^+^ ratio in the mitochondrial matrix. This could in turn inhibit the glycine decarboxylase complex, which is inhibited by NADH with a *K*_*i*_ of 15µM (Bykova *et al.*, 2014). Furthermore, the reductions in glutamate and glutamine pools together with the increase in α-ketoglutarate are most likely related to the reduced rates of ammonium production, from mitochondrial glycine oxidation, which would limit its re-fixation via the GS/GOGAT system. An important role of glutamate dehydrogenase (GDH) in this unbalanced ratio between α-ketoglutarate and glutamate has been discarded for two main reasons: (i) in the present scheme, NADH would be more available than NAD^+^, which thus would not support the catabolism of glutamate, and (ii) although it has been proposed that GDH could play a role in ammonium re-assimilation by mitochondria, the high *K*_*m*_ of this enzyme for NH_4_^+^ (in the range of a few mM) would not particularly favour the reductive amination of α-ketoglutarate. Additionally, an increase in the mitochondrial NADH/NAD^+^ ratio can contribute to inhibition of key reactions in the TCA cycle, particularly steps catalysed by the pyruvate dehydrogenase complex and isocitrate dehydrogenase (Bykova *et al.*, 2005), thereby limiting the turnover of the cycle. Consequently, a light-dependent limitation in TCA cycle turnover is generally observed in the light even though different mechanisms may be operating in high and low CO_2_ conditions. A partial TCA cycle in the light has previously been described by several investigations using different approaches (Sweetlove *et al.*, 2010; Tcherkez *et al.*, 2009). An alteration in mitochondrial photorespiratory reactions may also affect the chloroplasts, as limitation of glycine decarboxylation in barley reportedly affects the chloroplast redox state (Igamberdiev *et al.*, 2001). The ‘malate-valve’ probably plays an important role in linking metabolic processes between the cellular compartments (Scheibe, 2004), although additional systems seem to be active (Hebbelmann *et al.*, 2012).

The very low ^13^C labelling of citrate and α-ketoglutarate could be due to the existence of separate pools of these metabolites where a large fraction can be found in pools of low metabolic activity. Another possible explanation is that citrate and α-ketoglutarate are not directly produced from newly fixed carbon but comes from stored reserves as reported from experiments with *Brassica* by Gauthier *et al.*, (2010). However, the incorporation of ^13^C into several metabolites differed between our experiments and this report. For example, alanine and aspartate were very poorly labelled after 6h in the light in *Brassica* in air or low CO_2_ whereas we observed high labelling of these two metabolites already within our 2h experiment, both in high and low CO_2_ (Fig 6). The contrasting results can be due to differences between species and different experimental setups, including use of detached or attached leaves etc. The interactions between carbon and nitrogen metabolism is complex and additional detailed studies with different species and experimental systems will be needed to resolve this issue.

Interestingly, a higher incorporation of ^13^C label in succinate was observed in *mmdh1* plants compared to wild-type plants, at every time point, under both high and low CO_2_ (Table 1, Fig. 6B). Furthermore, the labelling was much higher than in citrate and α-ketoglutarate, and in low CO_2_ the succinate pool was also much larger in *mmdh1* plants than in wild-type plants. A possible explanation for these observations, illustrated in Fig. 6B, is that in *mmdh1* OAA to malate conversion is limited, which impairs malate/OAA exchange. In this situation cytosolic PEP carboxylase can fix H^13^CO_3_^−^ to form OAA, which can be reduced to malate by cytosolic MDH. This labelled malate could then be taken up by mitochondria and converted to succinate, which accumulate under these conditions. Therefore, it is tempting to propose a mechanism whereby succinate formation from malate via fumarate at the expense of FADH_2_ oxidation could perhaps relieve some of the limitation imposed on glycine oxidation by a high matrix redox state. Accordingly, it has been shown that an increase of α-ketoglutarate, a metabolite at the junction between the TCA cycle and nitrogen assimilation, can lead to activation of succinate dehydrogenase (Saito and Matsuda, 2010).

### The use of ^13^C unveils novel metabolic dynamics and regulation

The present study takes advantage of the potential to use ^13^C labelling to get additional information about plant metabolism. While analysis of pool sizes could identify a metabolic block in the glycine to serine conversion the labelling data gives further information about effects on the turnover of the TCA cycle. In addition, we found that sugars (more specifically hexoses/hexose phosphates and sucrose) rapidly became highly labelled following exposure to the tracer. Two other sugars, trehalose and maltose, had similar pool sizes and no further information about them would have been obtained using a conventional metabolomics approach. However, ^13^C labelling revealed that maltose had similar labelling kinetics to the other sugars, contrary to trehalose, which remained poorly labelled throughout the ^13^C treatment. This indicates that maltose is somehow a direct product of primary carbon incorporation (Fig. 6A). A similar observation, obtained by a different experimental system, has recently been reported by Szecowka *et al.* (2013). The cited authors proposed that the dramatic increase in labelled maltose is unlikely to originate from transient starch degradation, but rather from *de novo* biosynthesis of maltose in light. This is consistent with observations of very rapid ^14^CO_2_ labelling of maltose in spinach chloroplasts in earlier studies (Allen *et al.*, 2003; Bauwe *et al.*, 2010). Another interesting result is the accumulation of several amino acids, including aromatic and branched-chain amino acids, in the *mmdh1* mutant under low CO_2_. Surprisingly, however, the ^13^C incorporation in these metabolites was not correspondingly high, suggesting that their accumulation was due to transfers between pools rather than de novo biosynthesis (Fig. 6A). A similar reasoning may explain the low ^13^C incorporation in glutamine, glutamate and α-ketoglutarate (Fig. 6B). Indeed, the altered photorespiratory cycle in *mmdh1* certainly leads to an imbalanced ammonium refixation in the chloroplast. Furthermore, since glutamate, glutamine and α-ketoglutarate share the same carbon backbone, these metabolites probably only exchange carbon skeletons among their existing pools while some of the nitrogen accumulates in glycine. This recycling of backbone skeletons would limit the ^13^C incorporation into these three metabolites.

An obvious limitation in the present experimental setup lies in the fact that the CO_2_ concentration was changed simultaneously with the start of the ^13^C labelling. Consequently, as metabolite pools do change during the labelling period, metabolic fluxes cannot be properly calculated. Future prospects for the ^13^C estimations would therefore be to combine them with a flux model to estimate fluxes for the defined metabolic network. Recently an elegant method was published were non-stationary ^13^C flux analysis was used to monitor flux in Arabidopsis rosettes (Ma *et al.*, 2014). Furthermore, the experimental setup described in the present study has a limitation in time resolution, however, this may not be a problem when metabolic reactions downstream of the Calvin-Benson cycle are in focus. The report by Ma *et al.* (2014) showed that the metabolites involved in this cycle were saturated with label within ~15min whereas downstream metabolites were poorly labelled. Thus a longer labelling period would be needed to get reliable data. Another future prospect would be to include aspects of cell compartmentalization, which is necessary to fully elucidate metabolic fluxes and metabolism. The labelling results provide information about the rate of conversion of specific metabolites, which in turn can give hints on the existence of several subcellular pools. For example, the rather low labelling rate of malate, fumarate and succinate can be explained by the size and location of these pools in plant cells. The biggest fractions of these organic acids are located in the vacuole, where malate and fumarate can form temporary carbon sinks for photosynthate (Pracharoenwattana *et al.*, 2010; Keerberg *et al.*, 2011). Small, organellar pools with a rapid turnover will therefore be masked by bigger and less metabolically active pools. For such studies non-aqueous fractionation (Heise *et al.*, 2014) and protoplast fractionation (Keerberg *et al.*, 2011) procedures could be used in order to follow up leads from labelling experiments.

## Supplementary data

Supplementary data are available at *JXB* online.

Fig. S1. Labelling chamber.

Fig. S2. PCA score plot of Arabidopsis wild-type leaves from two experiments, one treated in high CO_2_ and one in low CO_2_.

Fig. S3. Y-predictive plots for chamber validation.

Fig. S4. Conceptual diagrams of ^13^C detection in (A) LC- and (B) GC-MS.

Fig. S5. Script output of ^13^C visualization plots, here data for sucrose under high CO_2_ in *mmdh1* and wild-type samples.

Fig. S6. Biological validation of the system and methodology by OPLS-DA of metabolic profiles of *mmdh1* and wild-type samples at high (A, B) and low CO_2_ (C, D).

Fig. S7. Glycine/serine ratios observed in wild type and *mmdh1* samples under (A) high, (B) ambient and (C) low CO_2_ conditions.

Fig. S8. Percentage ^13^C incorporated in hexose phosphates at high CO_2_ and low CO_2_ in *mmdh1* and wild-type samples.

Table S1. All MRM transitions and instrumental setup for LC-QqQ-MS analysis.

Table S2. List of metabolites analysed by GC-MS.

Table S3. Metabolite abundance under high and low CO_2_ treatment.

Table S4. Student’s *t*-test of the analysed metabolites in mmdh1.

Table S5. Student’s *t*-test of the analysed metabolites in wild type.

Table S6. Student’s *t*-test for the analysed metabolites.

Supplementary Data

## Abbreviations:

EI: electron impact
ESI: electrospray ionization
FADH_2_: flavin adenine dinucleotide (hydroquinone form)
GC: gas chromatography
GS/GOGAT: glutamine synthetase/ glutamine oxoglutarate aminotransferase
HILIC: hydrophilic interaction chromatography
LC: liquid chromatography
mMDH: mitochondrial malate dehydrogenase
m/z: mass-to-charge ratio
MRM: multiple-reaction-monitoring
MS: mass spectrometry
MS/MS: tandem mass spectrometry
NAD^+^: nicotinamide adenine dinucleotide (oxidized)
NADH: nicotinamide adenine dinucleotide (reduced)
NMR: nuclear magnetic resonance spectroscopy
OAA: oxaloacetate
OPLS: orthogonal projection to latent structures
OPLS-DA: OPLS-discriminant analysis
PCA: principal component analysis
PEP: phosphoenolpyruvate
QqQ: triple quadrupole
RNA: ribonucleic acid
RSD: relative standard deviation
Ru5P: ribulose-5-phosphate
Rubisco: ribulose bisphosphate carboxylase/oxygenase
TCA cycle: tricarboxylic acid cycle
TMS: trimethylsilyl
TOF: time of flight
UHPLC: ultra-high-performance liquid chromatography.
