# Supplementary material for: Reduced mitochondrial malate dehydrogenase activity has a strong effect on photorespiratory metabolism as revealed by 13C labelling
Source: J Exp Bot. 2016 Feb 17;67(10):3123–35. doi: 10.1093/jxb/erw030 (PMC4867893; doi:10.1093/jxb/erw030)
Supplement: Supplementary Data [file supp_67_10_3123__index.html]

Reduced mitochondrial malate dehydrogenase activity has a strong effect on photorespiratory metabolism as revealed by 13C labelling — Reduced mitochondrial malate dehydrogenase activity has a strong effect on photorespiratory metabolism as revealed by 13C labelling — Supplementary Data 

# Reduced mitochondrial malate dehydrogenase activity has a strong effect on photorespiratory metabolism as revealed by 13C labelling

## Supplementary Data

Data files

- supplementary\_figures\_S1\_S8\_Tables\_S1\_S6.pdf - Supplementary Data
