# Supplementary material for: Reduced mitochondrial malate dehydrogenase activity has a strong effect on photorespiratory metabolism as revealed by 13C labelling
Source: J Exp Bot. 2016 Feb 17;67(10):3123–35. doi: 10.1093/jxb/erw030 (PMC4867893; doi:10.1093/jxb/erw030)
Supplement: Supplementary Data [file supp_erw030_supplementary_figures_S1_S8_Tables_S1_S6.pdf]

Supplementary Data

A

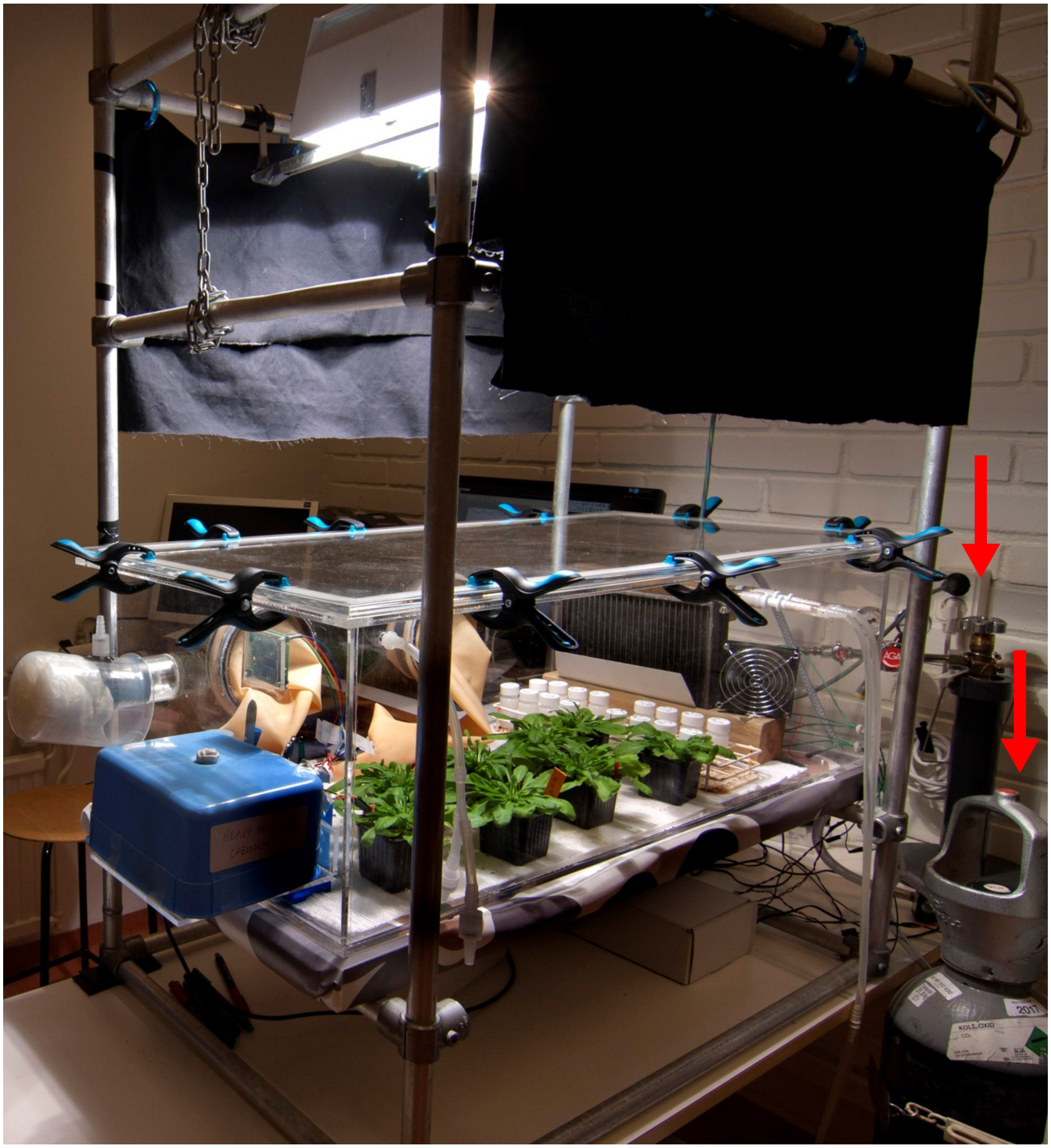

B

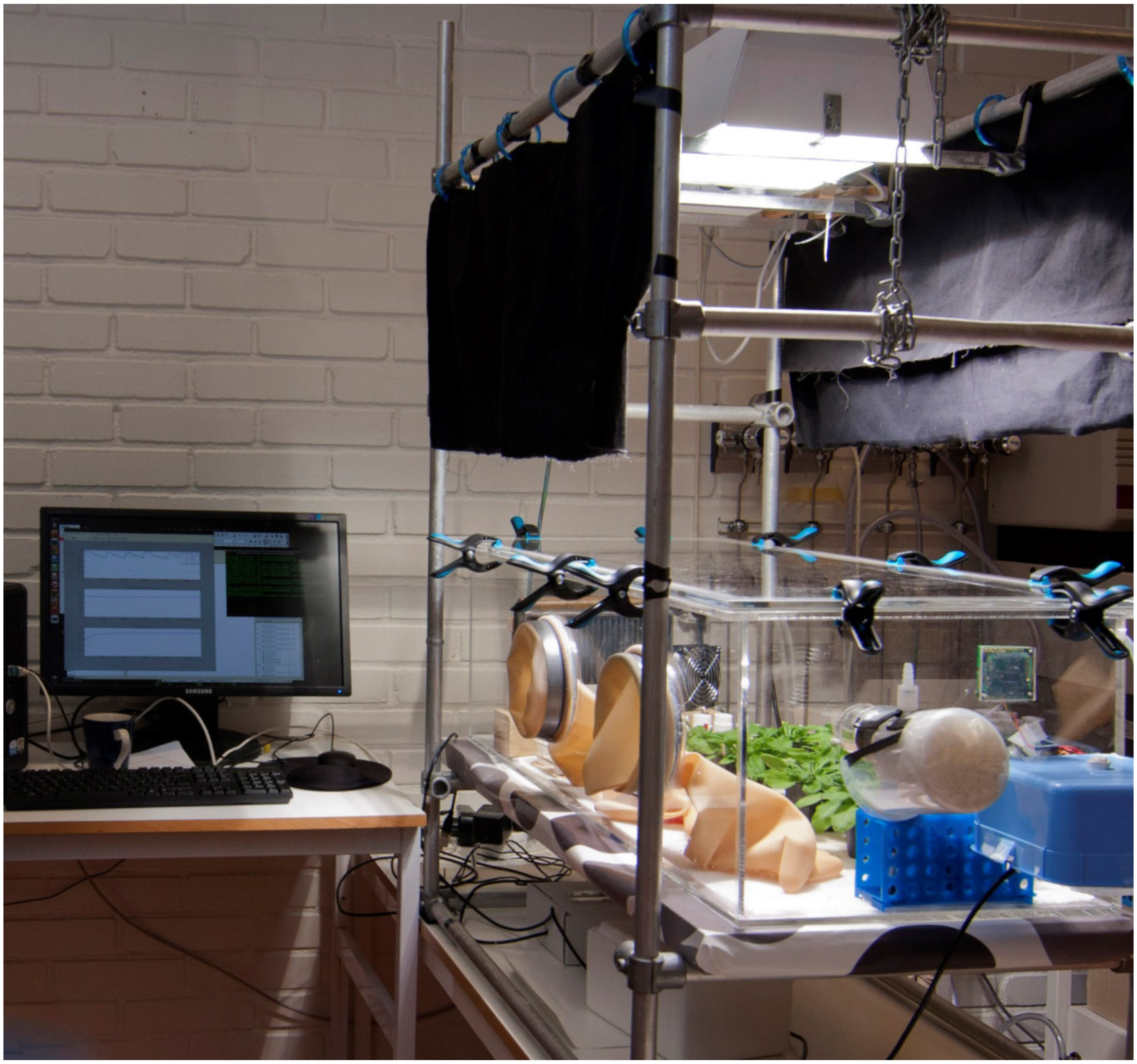

Suppl. Fig. 1 A) and B) Photos showing the Plexiglas labelling chamber with plants undergoing $^{13}CO_2$ labelling treatment. In A) the upper and lower red arrows indicate the small $^{13}CO_2$ bottle and the $^{12}CO_2$ bottle, respectively.

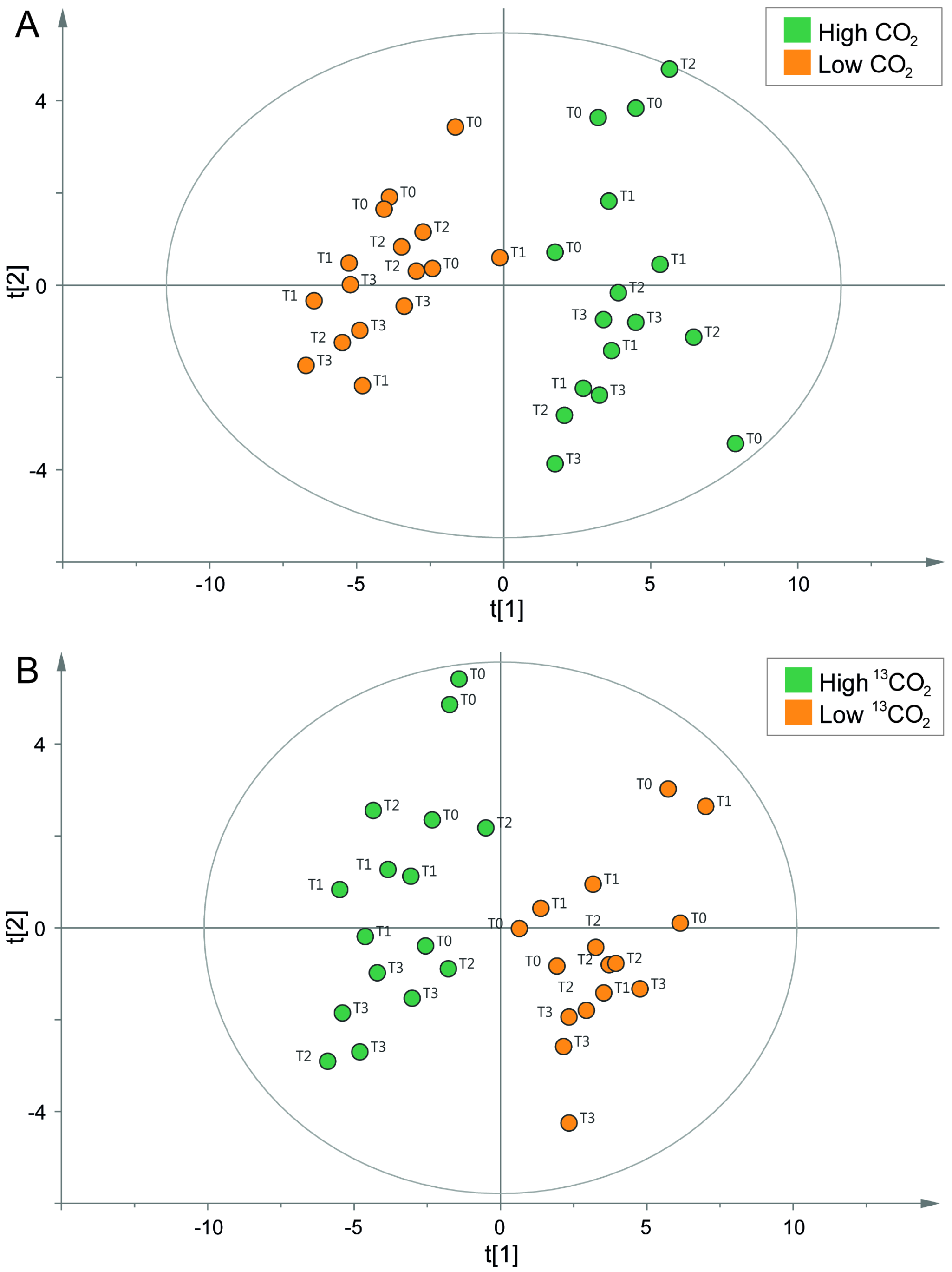

Suppl. Fig. 2 A) PCA score plot of Arabidopsis wild type leaves from two experiments, one in low CO_2 (orange) and one in high CO_2 (green). (PCA model: Components = 5, n = 32, R2X(cum) = 0.841, Q2 = 0.549). B) PCA score plot of Arabidopsis wild type leaves from two labelling experiments, one in low $^{13}CO_2$ (orange) and one in high $^{13}CO_2$ (green). (PCA model: Components = 4, n = 32, R2X(cum) = 0.688, Q2 = 0.369). (T0 = control, T1 = 30 min, T2= 60 min, T3 = 120 min of treatment). The first component (t[1]) describes the variation in X that can be predicted by Y and the second component (t_o[1]) explains the variation that is unique to X, in this case CO_2 concentration. Data were normalised to fresh weight (mg) and technical variation (t-score of internal standards).

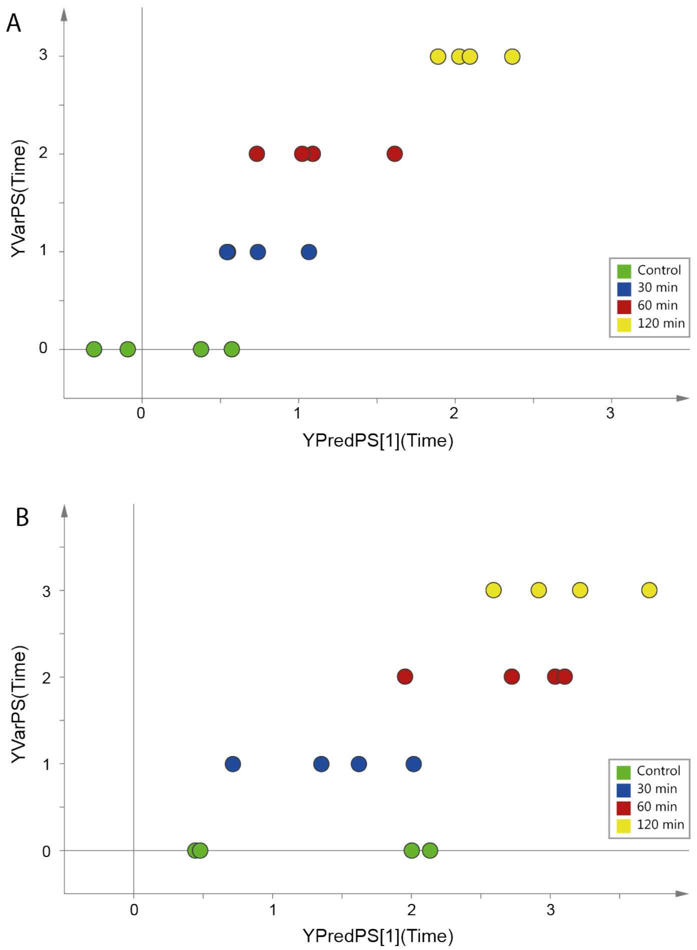

Suppl. Fig. 3 A) Y-predictive plot of the samples included in the experiment using CO_2, generated from a model based on the samples included in the experiment using $^{13}CO_2$, R2-value 0.95 (OPLS model: components = 1+6, n = 16, Y-var = time, X-var = 37, R2X(cum) = 0.801, R2Y(cum) = 0.999, Q2 = 0.888).

B) Y-predictive plot of the samples included in the experiment using $^{13}CO_2$, generated from a model based on the samples included in the experiment using CO_2, R2-value 0.61 (OPLS model: component = 1+2, n= 16, Y-var = time, X-var = 37, R2X(cum) = 0.539, R2Y(cum) = 0.954, Q2 = 0.835).

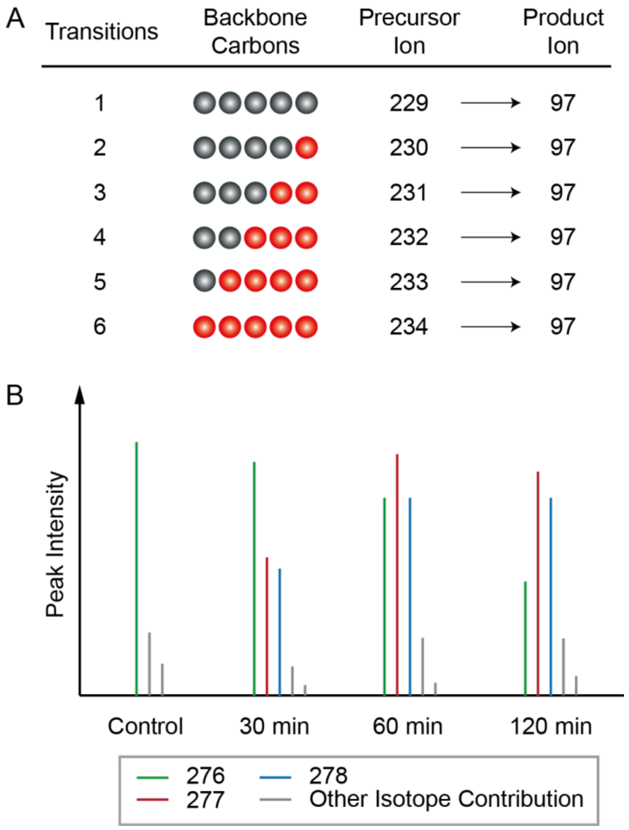

Suppl. Fig. 4 A) Conceptual diagram of ^{13}C detection in LC-MS, illustrating MRM transitions for the five carbon molecule ribulose-5-phosphate ($C_5H_{11}O_8P$, average mass 230.11 Da). The five carbons give six possible isotopes (precursor ions) when labelled, with masses ranging from 229 to 234, but the product ion 97 (PO_4H_2) is the same for all precursor ions. Gray and red dots represent ^{12}C and ^{13}C, respectively. B) Incorporation patterns for GC-MS data of glycine before labelling, and after 30, 60 and 120 min labelling. Glycine has two carbons, giving three possible isotopes: not labelled (green), one labelled carbon (red bars) or two labelled carbons (blue bars). Graphs show the shift of isotopes from unlabelled green in the control to labelled red and blue after two hours of ^{13}C treatment.

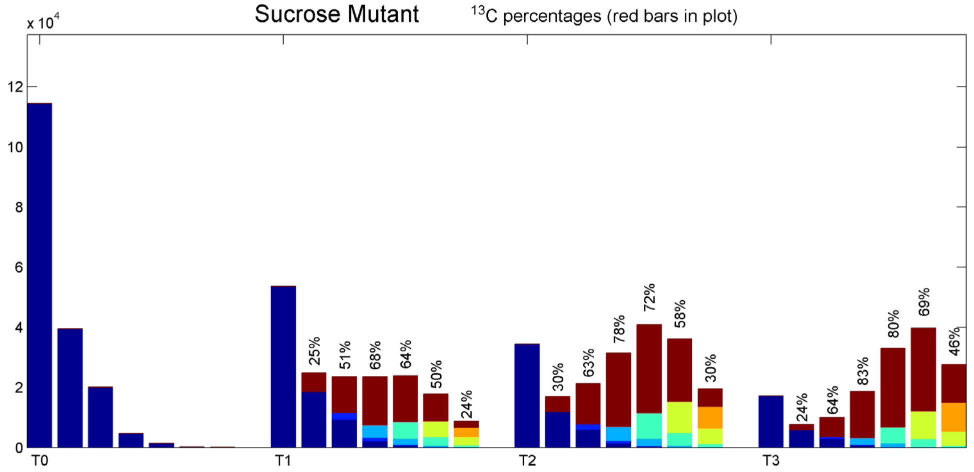

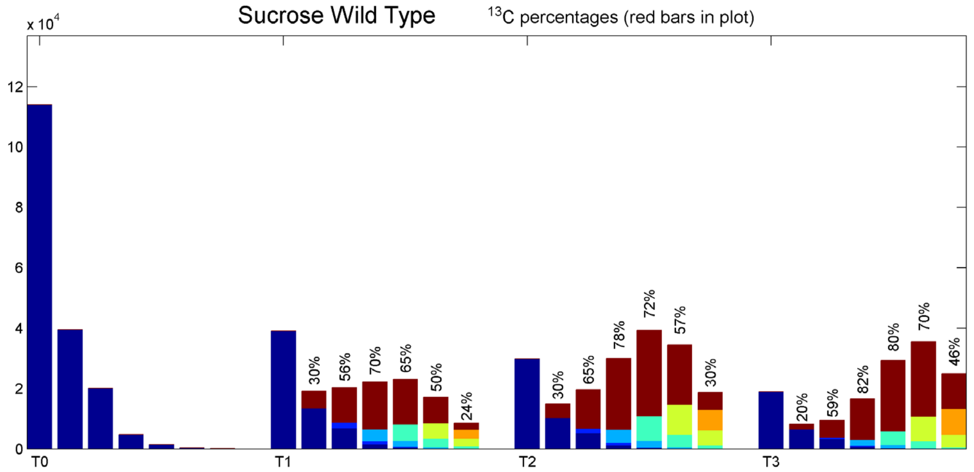

Suppl. Fig. 5 Script output of ^{13}C visualisation plots, here data for sucrose under high CO_2 in *mmdh1* (top) and wild type (bottom) samples. Since sucrose has six carbons, values for seven isotopes are calculated. Dark blue bars indicate unlabelled isotopes. The control, before start of treatment, is used as a reference for isotope distribution. The dark red bars indicate the ^{13}C introduced to the system in percent. The other colours represent the contributions from natural ^{13}C and the silica in the TMS-groups. The script generates one visualisation plot for every metabolite and a text file with the summed ^{13}C incorporation.

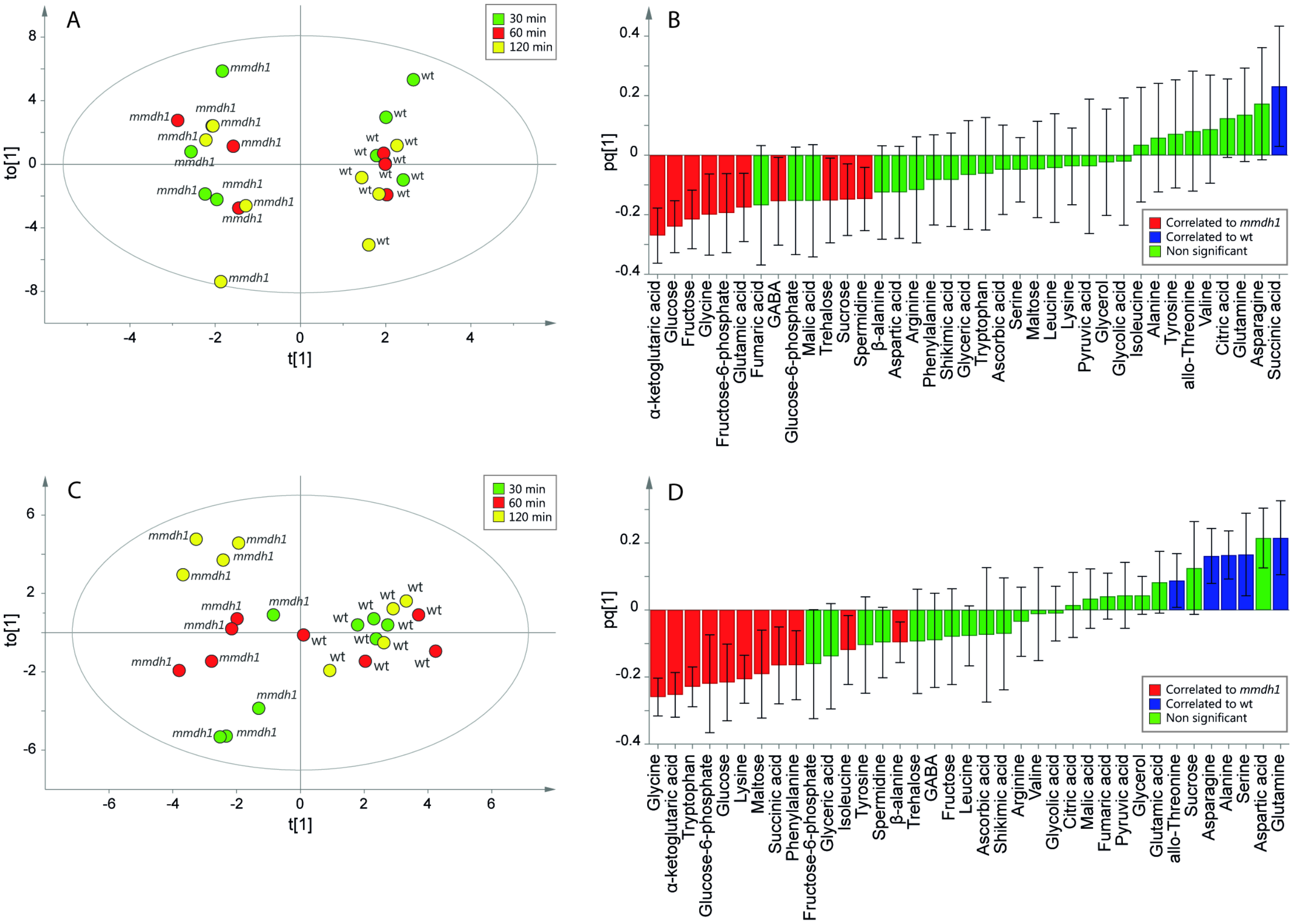

Suppl. Fig. 6 Biological validation of the system and methodology by OPLS-DA of metabolic profiles of *mmdh1* and wild type samples at high and low CO_2. (A) Score plot, high CO_2 (OPLS-DA model: components 1+3, n = 24, X-var = 37, R2X(cum) = 0.589, R2Y(cum) = 0.965, Q2 = 0.871). (B) loading plot, high CO_2. (C) Score plot, low CO_2 (OPLS-DA model: components 1+1, n = 24, X-var = 37, R2X(cum) = 0.337, R2Y(cum) = 0.860, Q2 = 0.709. (D) loading plot, low CO_2. Orthogonal separation of time points was observed for *mmdh1* samples under low CO_2, but no orthogonal separation was detected under high CO_2.

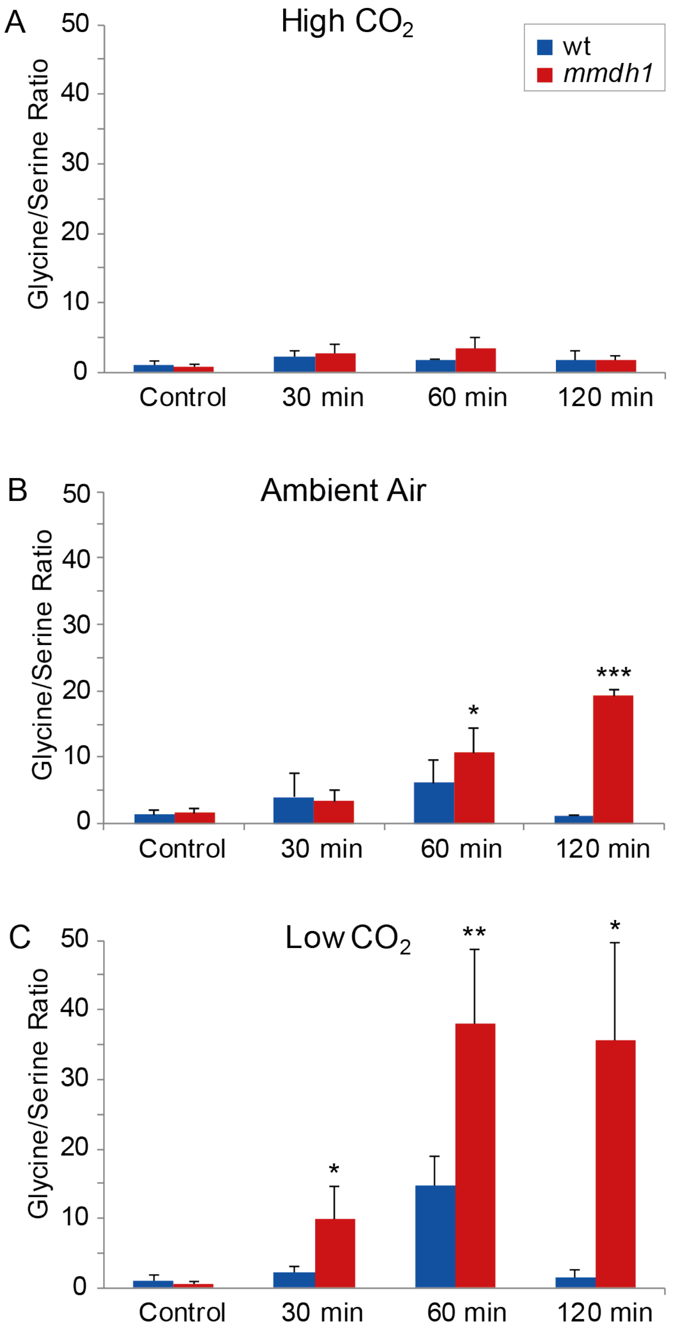

Suppl. Fig. 7 Glycine/serine ratios observed in wild type (blue) and *mmdh1* (red) samples under A) high (> 1000 µL L^{-1}) and B) Ambient air (400 µL L^{-1}) and C) low CO_2 (< 150 µL L^{-1}). The ratio was about ten times higher in the low CO_2 treatment than in the high CO_2 treatment, for both genotypes, but *mmdh1* showed a slower response than wild type. For the ambient air experiment wild type control and wild type 120 min (n = 3), otherwise n = 4. Statistical relevance was assessed by a Student's T-test with significance * = p < 0.05, ** = p < 0.01 and *** = p<0.001.

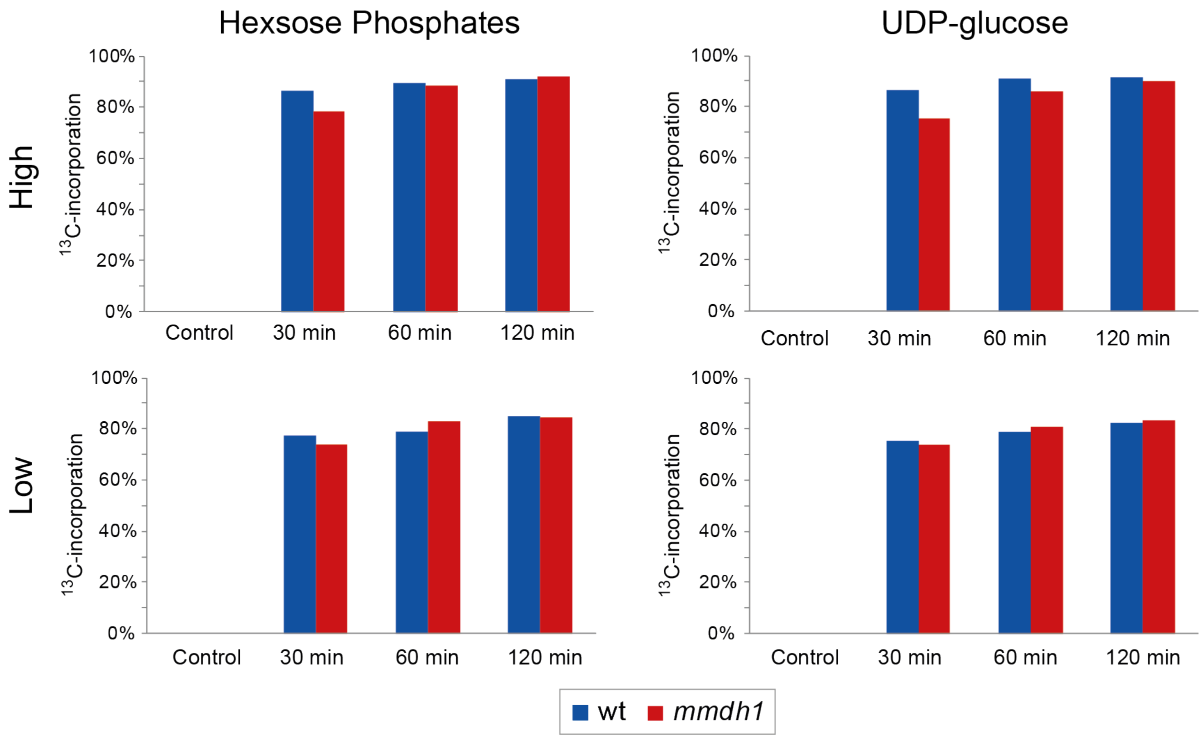

Suppl. Fig. 8 Percentage ^{13}C incorporated in hexose phosphates at high CO_2 and low CO_2 in *mmdh1* (red) and wild type (blue) samples. There was no significant difference in incorporation between genotypes. In high CO_2 conditions the sugar phosphates quickly became fully labelled (90 %). In low CO_2 conditions only 80 % of the carbons were labelled, indicating a lower incorporation rate.

Suppl. Table 1 All MRM transitions and instrumental setup for LC-QQQ-MS analysis. CE = Collision Energy; Cell Acc V = cell acceleration voltage.

Abbreviations for metabolites: 3PGA, 3-phosphoglyceric acid; 2PGA, 2-phosphoglyceric acid; 6PG, 6-phosphogluconic acid; ADP-glu, ADP-glucose; E4P, Erythrose-4-phosphate; F6P, Fructose-6-phosphate; G6P, Glucose-6-phosphate; G1P, Glucose-1-phosphate; FrubP, Fructose-1,6-bisphosphate; GAP, Glyceraldehyde-3-phosphate; DHAP, Dihydroxyacetone phosphate; PEP, Phosphoenolpyruvate; Ru5P, Ribulose-5-phosphate; X5P, Xylulose-5-phosphate; R5P, Ribose-5-phosphate; RubP, Ribulose-1,5-bisphosphate; S7P, Sedoheptulose-7-phosphate; T6P, Trehalose-6-phosphate; S6P, Sucrose-6-phosphate; UDP-glu, UDP-glucose.

Compound	Precursor Ion	Product Ion	Dwell	Fragmentor	CE	Cell Acc V	Polarity	Label
3PGA/2PGA	185	79	50	380	45	5	-	No
6PG	275	79	50	380	50	5	-	No
ADP-glu	588.1	79	50	380	55	5	-	No
E4P	199.1	97	50	380	5	5	-	No
F6P/G6P/G1P	259	97	50	380	15	5	-	No
FrubP	339	97	50	380	20	5	-	No
GAP/DHAP	169	97	50	380	5	5	-	No
PEP	167	79	50	380	10	5	-	No
Ru5P/X5P/R5P	229	97	50	380	10	5	-	No
RubP	309	97	50	380	35	5	-	No
S7P	289	97	50	380	20	5	-	No
T6P/S6P	421.1	79	50	380	50	5	-	No
UDP-glu	565	79	50	380	70	5	-	No
F6P_0	259	97	50	380	15	5	-	Yes
F6P_1	260	97	50	380	15	5	-	Yes
F6P_2	261	97	50	380	15	5	-	Yes
F6P_3	262	97	50	380	15	5	-	Yes
F6P_4	263	97	50	380	15	5	-	Yes
F6P_5	264	97	50	380	15	5	-	Yes
F6P_6	265	97	50	380	15	5	-	Yes
UDP-glu_0	565	79	50	380	70	5	-	Yes
UDP-glu_1	566	79	50	380	70	5	-	Yes
UDP-glu_2	567	79	50	380	70	5	-	Yes
UDP-glu_3	568	79	50	380	70	5	-	Yes
UDP-glu_4	569	79	50	380	70	5	-	Yes
UDP-glu_5	570	79	50	380	70	5	-	Yes
UDP-glu_6	571	79	50	380	70	5	-	Yes
UDP-glu_7	580	79	50	380	70	5	-	Yes

Suppl. Table 2 List of metabolites analysed by GC-MS. The Derivatization column lists the number of silyl (TMS) and methoxyamine groups (MeOX). Other columns: m0, Neutral fragment m/z; C Calc, the number of carbons analysed for the metabolite; RI, Retention Index; RT Window, column retention time window used to calculate the peak intensity.

Metabolite	Formula	Derivatization	Fragment Formula	m0	C Calc.	RI	RT Window
Glycolic acid	C2H4O3	2 TMS	C5H13O3Si2•	177	2	1073	329-333
Pyruvic acid	C3H4O3	2 TMS	C6H12NO3Si•	99	1	1048	316-319
Alanine	C3H7NO2	2 TMS	C6H16NO2Si2•	190	3	1100	343-345.5
Valine	C5H11NO2	2 TMS	C8H20NO2Si2•	218	2	1209	397-400
Glycerol	C3H8O3	3 TMS	C7H17O3Si2•	205	3	1261	419-425
Leucine	C6H13NO2	2 TMS	C8H20NSi•	158	4	1263	422.5-425
Isoleucine	C6H13NO2	2 TMS	C8H20NSi•	158	5	1284	431-436
Glycine	C2H5NO2	3 TMS	C10H26NO2Si3•	276	2	1301	437-443
Succinic acid	C4H6O4	2 TMS	C9H19O4Si2•	247	4	1306	442-445
Glyceric acid	C3H6O4	3 TMS	C10H24O4Si3	292	3	1318	445-450
Fumaric acid	C4H4O4	2 TMS	C9H17O4Si2•	245	4	1343	455-464
Serine	C3H7NO3	3 TMS	C9H22NO3Si3•	278	3	1349	459-461.7
Threonine	C4H9NO3	3 TMS	C12H30NO3Si3•	320	4	1375	469-473
beta- Alanine	C3H7NO2	3 TMS	C8H22NO2Si3•	248	3	1421	488-493
Malic acid	C4H6O5	3 TMS	C12H27O5Si3•	335	4	1476	508-515
Aspartic acid	C4H7NO4	3 TMS	C9H22NO2Si2•	232	3	1508	522-527
GABA	C4H9NO2	3 TMS	C12H30NO2Si3•	304	4	1524	528-533
a-Ketoglutaric acid	C5H6O5	2 TMS, 1 MeOX	C11H22NO4Si2•	198	5	1562	544-547
Glutamic acid	C5H9NO4	3 TMS	C10H24NO2Si2•	246	4	1607	558-564
Phenylalanine	C9H11NO2	2 TMS	C11H18NSi•	192	8	1624	567-569
Asparagine	C4H8N2O3	3 TMS	C9H23N2OSi2•	231	3	1659	577-581.5
Glutamine	C5H10N2O3	3 TMS	C9H19NO3Si2•	156	5	1763	614.5-617
Shikimic acid	C7H10O5	4 TMS	C8H20O2Si2•	204	4	1790	622-625
Citric acid	C6H8O7	4 TMS	C17H37O7Si4•	465	6	1803	626-630
Arginine	C6H14N4O2	5 TMS	C10H28N4Si2•	256	2	1813	630.7-632.2
Fructose	C6H12O6	5 TMS, 1 MeOX	C12H31O3Si3•	307	3	1857	642-645
Glucose	C6H12O6	5 TMS, 1 MeOX	C13H31O3Si3•	319	4	1881	649-654
Lysine	C6H14N2O2	3 TMS	C8H18NSi•	156	5	1912	661-663.5
Tyrosine	C9H11NO3	3 TMS	C8H20NO2Si2•	218	3	1930	667-670
Ascorbic acid	C6H8O6	4 TMS	C12H23O5Si3•	332	5	1931	668-671
Tryptophan	C11H12N2O2	6 TMS	C12H16NSi•	202	9	2213	748-751
Spermidine	C7H19N3	5 TMS	C8H18NSi•	156	5	2252	757.5-759.7
Fructose-6-phosphate	C6H13O9P	6 TMS, 1 MeOX	C11H21NO4Si3•	315	3	2286	766-769
Glucose-6-phosphate	C6H13O9P	6 TMS, 1 MeOX	C12H32O6PSi3•	387	3	2301	769.5-772.5
Sucrose	C12H22O11	8 TMS, 1 MeOX	C15H33O4Si3•	361	6	2619	846-851
Maltose	C12H22O11	8 TMS, 1 MeOX	C15H33O4Si3•	361	6	2712	870-872.2
Trehalose	C12H22O11	8 TMS, 1 MeOX	C15H33O4Si3•	361	6	2720	872-875

Suppl. Table 3 A) Metabolite abundance in high CO_2 treatment (>1000 µL L^{-1}) based on four biological replicates per sample. Averg. (average), stdev.(standard deviation). T0 (control), T1 (30 min treatment), T2 (60 min treatment) and T3 (120 min treatment).

	Avrg.	Stdev.	Avrg.	Stdev.	Avrg.	Stdev.	Avrg.	Stdev.	Avrg.	Stdev.	Avrg.	Stdev.	Avrg.	Stdev.	Avrg.	Stdev.
Treatment	high	high	high	high	high	high	high	high	high	high	high	high	high	high	high	high
Time point	T0	T0	T1	T1	T2	T2	T3	T3	T0	T0	T1	T1	T2	T2	T3	T3
Genotype	mutant	mutant	mutant	mutant	mutant	mutant	mutant	mutant	wt	wt	wt	wt	wt	wt	wt	wt
a-ketoglutaric acid	1975	300	2087	997	2579	817	1487	240	1414	299	1146	271	1167	261	897	134
Alanine	2115	958	2134	919	1996	1402	2032	934	2995	2391	2336	894	2233	882	2323	1324
Arginine	192	34	190	22	188	44	177	67	219	56	234	40	189	21	176	61
Ascorbic acid	1108	361	1292	473	1048	332	859	78	1177	292	1097	395	990	172	898	231
Asparagine	1201	147	1335	251	1191	203	1118	359	1463	164	1631	371	1422	85	1308	268
Aspartic acid	184286	14668	150126	12326	128192	19425	141273	19096	163730	19482	124229	13584	135452	7662	124846	29079
β-alanine	17992	1907	13132	3203	14175	2782	13461	1980	16813	2407	14740	798	15785	1857	13373	684
Citric acid	66384	19575	58939	21325	44185	22143	33138	16160	48383	1984	34553	8464	27271	7697	22190	7347
Fructose	1383263	147291	1650887	135615	1546252	78547	1710165	164029	1431894	115546	1484925	173289	1560626	109733	1503446	58512
Fumaric acid	2160	963	1481	384	1816	589	1270	358	1532	106	1339	393	1124	310	1021	445
GABA	232877	30665	232654	70843	205612	72135	190915	72505	187902	28123	172660	32590	118922	23775	119364	26746
Glucose	65988	9814	47878	7681	61968	13969	42664	10709	55297	9243	40327	6205	44873	6054	34961	11456
Glutamic acid	49695	5980	73778	15608	62463	7846	60084	20596	60861	14978	87597	19484	74207	4461	66328	12319
Glutamine	3268	642	1367	330	1518	486	1397	207	3118	978	1388	270	1351	146	1215	165
Glyceric acid	23769	5411	24598	5767	22218	7684	24392	4924	21230	6378	24059	6393	20893	1397	23092	5320
Glycerol	5819	4358	12854	7554	21408	4479	18023	10157	7145	4216	11157	4202	7643	901	11927	4829
Glycine	668	259	535	146	656	270	712	307	617	270	620	232	575	96	603	142
Glycolic acid	2091	475	2187	593	2376	822	2219	573	2122	580	2405	571	2438	604	2101	309
Isoleucine	1255	324	1427	322	1353	457	1358	425	1271	489	1489	383	1498	673	1344	273
Leucine	1178	85	1086	133	966	117	719	199	1064	181	988	144	968	206	717	248
Lysine	38497	5244	41290	3818	41893	3982	43364	5387	34063	4657	35784	4683	41417	3116	37941	5200
Malic acid	1604	430	1635	796	1828	696	1732	656	1793	534	1923	727	1298	479	1566	614
Maltose	3640	1046	4048	1291	4300	481	3159	1287	3563	900	3886	649	3618	983	2881	289
Phenylalanine	6825	1535	4522	789	6581	1888	10191	2086	6684	986	5214	1689	5687	790	8183	3158
Serine	7951	907	7910	1953	8707	2067	7146	1672	7776	1804	8666	1551	6610	1687	5975	1005
Shikimic acid	925	165	1020	113	927	228	971	358	676	155	931	227	765	72	719	163
Spermidine	3228	972	4031	977	5257	2780	3446	933	7899	2300	7778	3187	7036	953	5899	1998
Succinic acid	180859	33244	176246	7382	200803	16722	154239	5707	180438	12196	149325	22160	186581	27124	142820	21611
Sucrose	1840	342	1871	402	2031	566	1947	390	1961	223	2293	377	2030	565	1924	311
Threonine	1777	93	1728	357	1690	285	1655	56	1759	156	1637	281	1480	132	1459	132
Trehalose	809	109	1012	144	1031	153	913	478	730	131	917	114	1813	1651	1108	476
Tyrosine	813	304	872	211	743	264	667	292	822	361	886	233	879	226	791	174
Valine	948	196	901	54	996	133	1158	278	651	114	800	157	921	100	1003	123

Suppl. Table 3 B) Metabolite abundance in low CO_2 treatment (150 µL L^{-1}) based on four biological replicates per sample. Averg. (average), stdev.(standard deviation). T0 (control), T1 (30 min treatment), T2 (60 min treatment) and T3 (120 min treatment).

	Avrg.	Stdev.	Avrg.	Stdev.	Avrg.	Stdev.	Avrg.	Stdev.	Avrg.	Stdev.	Avrg.	Stdev.	Avrg.	Stdev.	Avrg.	Stdev.
Treatment	Low	Low	Low	Low	Low	Low	Low	Low	Low	Low	Low	Low	Low	Low	Low	Low
Time point	T0	T0	T1	T1	T2	T2	T3	T3	T0	T0	T1	T1	T2	T2	T3	T3
Genotype	mutant	mutant	mutant	mutant	mutant	mutant	mutant	mutant	wt	wt	wt	wt	wt	wt	wt	wt
a-ketoglutaric acid	1611	455	2878	298	15668	2988	17338	588	1020	263	753	203	1497	928	1128	184
Alanine	769	469	318	81	177	25	182	27	1037	213	421	172	467	233	230	48
Arginine	251	32	238	27	256	46	264	57	288	57	238	38	265	24	229	29
Ascorbic acid	1168	449	983	86	1160	180	1186	306	1169	258	1114	110	913	303	1153	288
Asparagine	171	33	174	45	199	19	205	33	226	78	163	12	148	47	163	34
Aspartic acid	119898	21136	36982	12942	7514	4202	7908	4585	119761	20155	40732	5774	46256	35604	38725	13933
β-alanine	547	31	672	136	555	99	637	87	468	80	496	162	465	116	635	51
Citric acid	20280	4496	15285	3676	16047	2560	13227	1282	16481	1762	15042	2749	15807	2034	13985	1913
Fructose	106962	15812	87606	13145	42192	10060	36233	13589	94882	34222	69926	13763	47060	34524	15471	4913
Fumaric acid	1096668	49679	1065692	193113	1151605	381213	1349496	136414	1309345	134296	1342324	76458	1312790	186914	1140969	246944
GABA	1483	496	1326	458	919	149	593	212	984	320	478	88	786	360	925	446
Glucose	314845	66854	291478	73340	255606	25962	203607	66631	264888	91722	219987	51769	157744	106207	112076	43676
Glutamic acid	49108	8533	33327	4798	22351	6427	8969	2124	42184	4316	13087	1607	31813	13344	34846	7702
Glutamine	36125	6307	36000	14760	18578	1202	11431	3755	56450	22043	83564	13039	52427	19991	37249	9981
Glyceric acid	2158	444	692	95	691	122	742	111	1952	211	591	84	665	97	605	152
Glycerol	12094	2921	8656	688	9460	2655	9246	1553	13296	1167	10557	1871	9945	3539	7981	2293
Glycine	2457	857	16286	5617	41964	8374	63301	2580	4672	2521	3591	1009	9754	6953	6028	2855
Glycolic acid	367	25	348	22	395	57	375	28	481	226	356	52	378	42	360	58
Isoleucine	837	162	1134	626	1323	318	2381	705	1171	193	1314	507	1253	380	785	257
Leucine	658	199	886	860	1435	529	3313	887	881	189	1057	603	896	390	390	153
Lysine	1160	200	1849	1485	1694	145	3750	556	1248	197	1054	345	1042	398	790	173
Malic acid	28162	3095	33074	4122	31120	3389	36913	4922	29914	3124	33565	6069	39254	9437	30121	5010
Maltose	2348	602	1655	569	1762	311	1657	751	2135	821	1410	635	1057	505	1341	303
Phenylalanine	2906	634	2716	1008	3676	488	5661	731	3483	988	2849	906	3269	1646	2041	188
Serine	3880	359	1810	612	1170	351	1986	759	4623	810	1589	305	3687	2318	4087	1091
Shikimic acid	8720	1496	7271	3120	7922	1638	5452	2096	8211	2119	6365	1992	5792	1528	7351	1504
Spermidine	773	207	614	123	708	112	810	141	929	273	689	114	660	240	567	89
Succinic acid	1495	126	2034	840	5531	2209	13150	3582	5350	662	3470	950	3768	2100	2875	736
Sucrose	290495	29748	161266	26756	100137	22774	55919	12862	308772	58969	204325	18807	147670	40139	92361	3482
Threonine	1210	106	1251	224	1411	284	1322	115	1586	80	1452	264	1646	516	1452	538
Trehalose	1746	136	1382	128	1363	234	1222	71	1613	223	1351	125	1249	197	1229	171
Tyrosine	615	74	2456	3475	1929	502	4630	2921	909	210	1940	2014	1331	694	720	80
Valine	392	135	342	192	296	101	592	332	501	80	498	238	403	142	204	67

Suppl. Table 4. Student's t-test of the analysed metabolites in *mmdh1*. The test done between the control (T0) and the time points sampled during treatment (T1, T2 and T3).

mmdh1 **p< 0.02**	**High** **T0 to T1**	**High** **T0 to T2**	**High** **T0 toT3**	**Low** **T0 to T1**	**Low** **T0 to T2**	**Low** **T0 toT3**
α-ketoglutaric acid	NS	NS	signf	signf	signf	signf
Alanine	NS	NS	NS	NS	NS	NS
Arginine	NS	NS	NS	NS	NS	NS
Ascorbic acid	NS	NS	NS	NS	NS	NS
Asparagine	NS	NS	NS	NS	NS	NS
Aspartic acid	signf	signf	NS	signf	signf	signf
B-alanine	NS	NS	NS	NS	NS	NS
Citric acid	NS	NS	NS	NS	NS	NS
Fructose	NS	NS	signf	NS	signf	signf
Fumaric acid	NS	NS	NS	NS	NS	NS
GABA	NS	NS	NS	NS	NS	NS
Glucose	NS	NS	NS	NS	NS	NS
Glutamic acid	NS	NS	signf	NS	NS	signf
Glutamine	NS	signf	NS	NS	signf	signf
Glyceric acid	signf	signf	signf	signf	signf	signf
Glycerol	NS	NS	NS	NS	NS	NS
Glycine	NS	NS	NS	signf	signf	signf
Glycolic acid	NS	NS	NS	NS	NS	NS
Isoleucine	NS	NS	NS	NS	NS	NS
Leucine	NS	NS	NS	NS	NS	signf
Lysine	NS	NS	signf	NS	signf	signf
Malic acid	NS	NS	NS	NS	NS	NS
Maltose	NS	NS	NS	NS	NS	NS
Phenylalanine	NS	NS	NS	NS	NS	signf
Serine	NS	NS	NS	signf	signf	NS
Shikimic acid	NS	NS	NS	NS	NS	NS
Spermidine	NS	NS	NS	NS	NS	NS
Succinic acid	NS	NS	NS	NS	NS	signf
Sucrose	NS	NS	NS	signf	signf	signf
Threonine	NS	NS	NS	NS	NS	NS
Trehalose	NS	NS	NS	NS	NS	signf
Tyrosine	NS	NS	NS	NS	signf	NS
Valine	NS	NS	NS	NS	NS	NS

Suppl. Table 5. Student's t-test of the analysed metabolites in wild type. The test done between the control (T0) and the time points sampled during treatment (T1, T2 and T3).

**Wild type**<br>**p< 0.02**	**High**<br>**T0 to T1**	**High**<br>**T0 to T2**	**High**<br>**T0 toT3**	**Low**<br>**T0 to T1**	**Low**<br>**T0 to T2**	**Low**<br>**T0 toT3**
α-ketoglutaric acid	NS	NS	NS	NS	NS	NS
Alanine	NS	NS	NS	signf	signf	signf
Arginine	NS	NS	NS	NS	NS	NS
Ascorbic acid	NS	NS	NS	NS	NS	NS
Asparagine	NS	NS	NS	NS	NS	NS
Aspartic acid	NS	NS	NS	signf	NS	signf
β-alanine	NS	NS	NS	NS	NS	NS
Citric acid	NS	NS	NS	NS	NS	NS
Fructose	NS	signf	signf	NS	NS	signf
Fumaric acid	NS	NS	NS	NS	NS	NS
GABA	NS	NS	NS	NS	NS	NS
Glucose	NS	signf	NS	NS	NS	NS
Glutamic acid	NS	NS	NS	signf	NS	NS
Glutamine	NS	NS	NS	NS	NS	NS
Glyceric acid	NS	NS	signf	signf	signf	signf
Glycerol	NS	NS	NS	NS	NS	signf
Glycine	NS	NS	signf	NS	NS	NS
Glycolic acid	NS	NS	NS	NS	NS	NS
Isoleucine	NS	NS	NS	NS	NS	NS
Leucine	NS	NS	NS	NS	NS	NS
Lysine	NS	NS	NS	NS	NS	NS
Malic acid	NS	signf	NS	NS	NS	NS
Maltose	NS	NS	NS	NS	NS	NS
Phenylalanine	NS	NS	NS	NS	NS	NS
Serine	NS	NS	NS	signf	NS	NS
Shikimic acid	NS	NS	NS	NS	NS	NS
Spermidine	NS	NS	NS	NS	NS	NS
Succinic acid	NS	NS	NS	NS	NS	signf
Sucrose	NS	NS	NS	NS	signf	signf
Threonine	NS	NS	NS	NS	NS	NS
Trehalose	NS	NS	NS	NS	NS	NS
Tyrosine	NS	NS	NS	NS	NS	NS
Valine	NS	NS	NS	NS	NS	signf

Suppl. Table 6. Student's t-test for the analysed metabolites. The two genotypes was compared at each time point in two treatments, high CO_2 to the left and low CO_2 to the right.

**mmdh-wt Significance p = 0.02**	**High T0 Signf.**	**High T1 Signf.**	**High T2 Signf.**	**High T3 Signf.**	**Low T0 Signf.**	**Low T1 Signf.**	**Low T2 Signf.**	**Low T3 Signf.**
α-ketoglutaric acid	NS	NS	NS	signf	NS	signf	signf	signf
Alanine	NS	NS	NS	NS	NS	NS	NS	NS
Arginine	NS	NS	NS	NS	NS	NS	NS	NS
Ascorbic acid	NS	NS	NS	NS	NS	NS	NS	NS
Asparagine	NS	NS	NS	NS	NS	NS	NS	NS
Aspartic acid	NS	NS	NS	NS	NS	NS	NS	signf
β-alanine	NS	NS	NS	NS	NS	NS	NS	NS
Citric acid	NS	NS	NS	NS	NS	NS	NS	NS
Fructose	NS	NS	NS	NS	NS	NS	NS	NS
Fumaric acid	NS	NS	NS	NS	NS	NS	NS	NS
GABA	NS	NS	NS	NS	NS	NS	NS	NS
Glucose	NS	NS	NS	NS	NS	NS	NS	NS
Glutamic acid	NS	NS	NS	NS	NS	signf	NS	signf
Glutamine	NS	NS	NS	NS	NS	signf	NS	NS
Glyceric acid	NS	NS	NS	NS	NS	NS	NS	NS
Glycerol	NS	NS	NS	NS	NS	NS	NS	NS
Glycine	NS	NS	signf	NS	NS	signf	signf	signf
Glycolic acid	NS	NS	NS	NS	NS	NS	NS	NS
Isoleucine	NS	NS	NS	NS	NS	NS	NS	NS
Leucine	NS	NS	NS	NS	NS	NS	NS	signf
Lysine	NS	NS	NS	NS	NS	NS	NS	signf
Malic acid	NS	NS	NS	NS	NS	NS	NS	NS
Maltose	NS	NS	NS	NS	NS	NS	NS	NS
Phenylalanine	NS	NS	NS	NS	NS	NS	NS	signf
Serine	NS	NS	NS	NS	NS	NS	NS	NS
Shikimic acid	NS	NS	NS	NS	NS	NS	NS	NS
Spermidine	NS	NS	NS	NS	NS	NS	NS	signf
Succinic acid	NS	NS	NS	NS	signf	signf	NS	signf
Sucrose	NS	NS	NS	NS	NS	signf	NS	signf
Threonine	NS	NS	NS	NS	signf	NS	NS	NS
Trehalose	NS	NS	NS	NS	NS	NS	NS	NS
Tyrosine	NS	NS	NS	NS	NS	NS	NS	NS
Valine	NS	NS	NS	NS	NS	NS	NS	NS
